# Alismatis Rhizoma prevents AβO-induced neuronal cell death and synaptic loss in Alzheimer’s disease models

**DOI:** 10.3389/fphar.2026.1898356

**Published:** 2026-09-04

**Authors:** Seungmin Lee, In Gyoung Ju, Minji Lee, Jin Hee Kim, Yujin Choi, Chanmi Baek, Chan Kim, Kyuho Moon, Minho Moon, Yong Jun Kim, Myung Sook Oh

**Affiliations:** 1 Department of Biomedical and Pharmaceutical Sciences, Graduate School, Kyung Hee University, Seoul, Republic of Korea; 2 Department of Oriental Pharmaceutical Science, College of Pharmacy, Kyung Hee University, Seoul, Republic of Korea; 3 Department of Precision Medicine, Graduate School, Kyung Hee University, Seoul, Republic of Korea; 4 Department of Integrated Drug Development and Natural Products, Graduate School, Kyung Hee University, Seoul, Republic of Korea; 5 Department of Biochemistry, College of Medicine, Konyang University, Daejeon, Republic of Korea; 6 Department of Pathology, College of Medicine, Kyung Hee University, Seoul, Republic of Korea

**Keywords:** Alismatis Rhizoma, BDNF-related signaling, hippocampal plasticity, neuroprotection, PI3K/Akt/GSK-3β signaling

## Abstract

**Background:**

Alzheimer’s disease (AD), the most prevalent form of dementia, is a neurodegenerative disease characterized by abnormal accumulation of amyloid-β (Aβ), which leads to memory impairment, synaptic dysfunction, and neuronal loss. This study investigated whether Alismatis Rhizoma (AR) could prevent Aβ oligomer (AβO)-induced synaptic dysfunction, neuronal cell death, and consequent memory decline.

**Methods:**

For *in vitro* studies, HT22 cells and human embryonic stem cells (hESC)-derived hippocampal neurons exposed to AβO were treated with AR. For *in vivo* studies, we administrated AR orally at 50 and 200 mg/kg to mice intrahippocampally injected with AβO and memory function was assessed using behavioral tests. Cellular and hippocampal tissue samples were analyzed by Western blotting and immunostaining.

**Results:**

In AβO-injected mice, AR, particularly at 200 mg/kg, attenuated memory deficits, reduced hippocampal neuronal degeneration, preserved synaptic protein immunoreactivity and increased markers of hippocampal cell proliferation and immature neurons. These effects were accompanied by changes in PI3K/Akt/GSK-3β and ERK/CREB signaling, increased mature BDNF levels, and reduced cleaved caspase-3 levels. AR at 30 and 300 μg/mL protected HT22 cells and hESC-derived hippocampal neurons against AβO-induced loss of viability, while 300 μg/mL AR additionally reduced cleaved caspase-3 levels and increased inhibitory GSK-3β Ser9 phosphorylation in HT22 cells.

**Conclusion:**

Collectively, AR attenuated AβO-induced neuronal and synaptic injury and the associated memory impairment in acute experimental models. These findings provide promising preclinical evidence for the neuroprotective potential of AR and support its further evaluation in chronic and progressive AD models.

## 1 Introduction

Alzheimer’s disease (AD) is a representative neurodegenerative disorder and the most prevalent form of dementia, responsible for 60%–70% of all cases (Hampel et al., 2025). Its prevalence continues to increase with global population aging, creating a major public health burden (Breijyeh and Karaman, 2020). Clinically, AD presents with gradual memory decline, language dysfunction, and altered sleep patterns (Abdulkhaliq et al., 2026). In patients with AD, brain atrophy and ventricular enlargement are commonly observed, along with aberrant protein aggregates, including amyloid-β (Aβ) deposits and neurofibrillary tangles (Liu et al., 2024).

Aβ is generated through amyloidogenic processing of amyloid precursor protein and subsequently aggregates into oligomers, protofibrils, and plaques (Ahmadi et al., 2025). Among these species, soluble Aβ oligomers (AβO) are considered particularly neurotoxic because they disrupt synaptic function before extensive plaque deposition occurs (Lista et al., 2024). In the brain, soluble AβOs disrupt synaptic integrity and plasticity, thereby contributing to neuronal injury and memory impairment (Behroozi et al., 2026). Moreover, AβO reduces the levels of neurotrophic factors including brain-derived neurotrophic factor (BDNF), which are vital for memory and synaptic function. This decline contributes to a vicious cycle that exacerbates AD pathology (Gao et al., 2022). As AβO plays a key role in AD pathogenesis, protecting hippocampal neurons and synapses from AβO-mediated injury could be a potential strategy to prevent the disease.

Alismatis Rhizoma [AR; rhizome parts of *Alisma orientale* (Sam.) Juzep. (Alismataceae)], is traditionally employed to manage edema, promote diuresis, and regulate water metabolism (Tian et al., 2014). Pharmacological studies have reported anti-inflammatory, anticancer, hypolipidemic, and hypoglycemic activities of AR (Dai et al., 1991; Fong et al., 2007; Dan et al., 2011; Li and Qu, 2012). More recently, Zhan et al. (2024) demonstrated that AR alleviated memory deficits in mice with high-fat diet-induced obesity by reducing neuroinflammation and neuronal injury and improving glymphatic function. These findings suggest that AR may exert neuroprotective effects beyond its traditional indications (Zhan et al., 2024). However, whether AR can protect hippocampal neurons and synapses against AβO-induced injury remains unclear. Therefore, the present study aimed to determine whether AR could attenuate AβO-induced neuronal and synaptic injury and the associated memory impairment using AβO-exposed HT22 cells, human embryonic stem cell (hESC)-derived hippocampal neurons, and mice receiving an intrahippocampal AβO injection. We also examined signaling changes associated with neuronal survival, apoptosis, and synaptic plasticity.

## 2 Materials and methods

### 2.1 Preparation and standardization of AR

AR was obtained from Jung Do Herbal Drug Company (Seoul, Republic of Korea), and a voucher specimen (BON24071001) was deposited in the herbarium of the College of Pharmacy, Kyung Hee University (Seoul, Republic of Korea). To prepare the water extract, dried AR rhizomes were extracted by boiling in distilled water for 2 h, followed by filtration and lyophilization. The powder was obtained at a yield of 21.25%, and stored at 4 °C.

Alisol A (AA) and alisol B 23-acetate (AB) in AR were quantified via High-performance liquid chromatography with ultraviolet detection (HPLC-UV). Methanol stock solutions (1.0 mg/mL) of standards were diluted to yield a calibration range of 3.91–250 μg/mL. AR extracts (5.0 mg/mL in distilled water) were sonicated for 10 min, and all solutions were filtered through a 0.22 μm PTFE filter before analysis. Chromatographic separation was performed on an Agilent Eclipse Plus C18 column (4.6 mm × 100 mm, 3.5 μm) at a flow rate of 0.4 mL/min, using 0.1% aqueous formic acid (A) and acetonitrile (B) as the mobile phase. The gradient elution profile was: 80% B (0–20 min), 80%–100% B (20–30 min), 100% B (30–40 min), and 80% B (40–41 min). The injection volume was 5.0 μL, with detection at 210 nm. Samples were analyzed in quadruplicate.

### 2.2 Preparation of AβO solution

Soluble Aβ oligomers were prepared with minor modifications to a previously reported method (Ju et al., 2024). Aβ_1–42_ peptide (AnaSpec, Fremont, CA, USA) was dissolved in HFIP at 1 mg/mL and incubated at room temperature for 72 h. The solution was aliquoted and vacuum-dried to obtain Aβ_1–42_ monomer films, which were then reconstituted in DMSO to generate a 1 mM stock solution. To induce oligomerization, the stock solution was diluted to 50 μM in DMEM/F12 medium (Welgene, Gyeongsan, Republic of Korea) and maintained at 4 °C for 24 h. The same peptide lot was used for all experiments to reduce batch-related variability.

### 2.3 Animals and treatment

Male ICR mice (6 weeks old; Daehan Biolink, Eumseong, Republic of Korea) were maintained under controlled conditions (23 °C ± 1 °C, 60% ± 10% humidity, 12 h light/dark cycle) with free access to food and water. After a 1-week acclimatization period, mice were used for experiments. ICR mice were selected as a commonly used outbred strain for neurobehavioral and pharmacological studies of Aβ-induced cognitive impairment (Ju et al., 2025). All experimental procedures followed the principles described in the Guide for the Care and Use of Laboratory Animals, 8th edition (National Research Council, 2011).

### 2.4 Stereotaxic surgery procedure and animal study design

Mice were anesthetized and positioned on a stereotaxic instrument (myNeuroLab, St. Louis, MO, United States) to administer intrahippocampal injections of AβO (50 μM, 3 μL) into the right hippocampus. The solution was delivered into the target site at a flow rate of 0.5 μL/min. The injection needle was positioned using bregma-referenced coordinates: AP −2.0 mm, ML +1.5 mm, and DV −2.0 mm [15]. For the sham group, mice were injected with vehicle only, using the same injection volume as the AβO-treated groups. The accuracy of stereotaxic injection was confirmed by observing the needle path in brain slices.

A total of 62 male ICR mice were randomly divided into the following five groups: (1) sham group (sham-operated and orally administered 1 × saline; n = 13), (2) AβO group (AβO-injected and orally administered 1 × saline; n = 13), (3) AβO + AR 50 mg/kg group (AβO-injected and orally administered AR at 50 mg/kg/day; n = 13), (4) AβO + AR 200 mg/kg group (AβO-injected and orally administered AR at 200 mg/kg/day; n = 13), and (5) AβO + donepezil (DNZ) group (AβO-injected and orally administered DNZ at 2 mg/kg/day; n = 10). AR was prepared in 1× saline and given by oral gavage daily for a total of 17 days. On the third day of AR administration, AβO was injected into the hippocampus. DNZ (Sigma-Aldrich, St. Louis, MO, United States) was used as a positive control (Kim et al., 2014).

### 2.5 Novel objective recognition test (NORT)

The NORT was conducted as previously described (Eo et al., 2022). NORT was conducted on day 14 of AR treatment using a dark open-field arena (45 × 45 × 45 cm). In the familiarization phase, each mouse was placed in the arena with two identical objects for 4 min. Twenty-four hours later, the animal was reintroduced to the same arena, in which one familiar object had been exchanged for a new object, and exploratory behavior was recorded for 4 min. Only direct object-directed behavior, such as facing, sniffing, or contacting the object, was counted as exploration. Novel object preference was calculated as follows: novel object exploration time/(novel object exploration time + familiar object exploration time) × 100. Exploration times during the NORT were independently recorded by two observers blinded to the experimental groups. The final exploration time for each object was calculated as the mean of the two independent measurements for each animal.

### 2.6 Passive avoidance test (PAT)

The PAT was conducted according to a previously described method, with minor modifications (Lee et al., 2025). PAT was performed on day 16 of AR administration using a shuttle box consisting of equal-sized light and dark compartments (21 × 21 × 21 cm). In the acquisition phase, mice received a foot shock (0.75 mA, 3 s) after completely entering the dark chamber. The retention phase was conducted 24 h later without shock, and step-through latency was measured up to 300 s.

### 2.7 Brain tissue preparation

For tissue preparation, mice were anesthetized with 2,2,2-tribromoethanol (25 μL/g, i.p.) and transcardially perfused with 0.05 M PBS. Brains were fixed in 4% paraformaldehyde, post-fixed overnight at 4 °C, and cryoprotected in 30% sucrose/PBS. Coronal brain sections were prepared at 25 μm thickness using a freezing sliding microtome and stored at 4 °C in cryoprotectant solution until use.

### 2.8 FJB staining

Slide-mounted brain sections were processed for FJB staining using potassium permanganate pretreatment followed by incubation with 0.002% FJB in 0.1% acetic acid for 30 min. Sections were mounted with DPX and imaged using a K1-Fluo confocal microscope (Nanoscope Systems, Daejeon, Republic of Korea. Three hippocampal sections per mouse, covering anterior, middle, and posterior levels, were analyzed with ImageJ, and the mean number of FJB-positive cells was used for statistical analysis. Histological quantification was performed by an investigator blinded to group allocation.

### 2.9 Immunohistochemistry

Synaptic marker immunohistochemistry was performed on free-floating brain sections. Sections were treated with 1% H_2_O_2_, blocked with serum- and BSA-containing buffer, and incubated with primary antibodies overnight at 4 °C. After reaction with biotinylated secondary antibodies, signals were amplified using the Vectastain Elite ABC Kit and visualized with DAB. SYP (1:500, S5768, Sigma-Aldrich) and PSD-95 (1:1,000, AB18258, Abcam) immunoreactivities were separately quantified as optical density in the CA3 stratum lucidum and dentate gyrus molecular layer (DG-ML) using image analysis software.

For BDNF immunofluorescence, sections were incubated with an anti-BDNF antibody (1:500, AB108319, Abcam) overnight at 4 °C, followed by a fluorophore-conjugated secondary antibody and DAPI counterstaining. Images were obtained using a K1-Fluo confocal microscope (Nanoscope Systems, Daejeon, Republic of Korea).

For immunofluorescence analysis, brain sections were incubated overnight at 4 °C with the following primary antibodies: DCX (1:250, SC-8066, Santa Cruz) and Ki67 (1:200, AB15580, Abcam). Following washing, the sections were incubated with appropriate fluorescent secondary antibodies (1:500, Invitrogen) for 1 h at RT. After several washes with PBS, sections were stained with DAPI for 20 min. Fluorescence images were acquired with a K1-Fluo confocal microscope (Nanoscope Systems, Daejeon, Republic of Korea). The number of DCX- and Ki67-positive cells in the DG region of the hippocampus was quantified by stereological counting using ImageJ software. Histological quantification was performed by an investigator blinded to group allocation.

### 2.10 Western blot analysis

Western blotting was conducted according to a previously described method (Choi et al., 2023). The samples were extracted with inhibitor-containing RIPA buffer and quantified by the Bradford assay. Equal protein amounts were separated by SDS-PAGE, transferred to PVDF membranes, blocked, and probed with primary antibodies overnight at 4 °C. After incubation with HRP-conjugated secondary antibodies, bands were detected by ECL, imaged using a ChemiDoc XRS + system, and quantified with ImageJ. The following primary antibodies were used: β-actin (1:2,000, SC-47778HRP, Santa Cruz), cAMP response element-binding protein (CREB; 1:1,000, 06-863, Millipore), p-CREB (1:1,000, #9198, Cell signaling), p44/42 MAPK (ERK; 1:2000, SC-93, Santa Cruz), BDNF (1:2000, AB108319, Abcam), phosphatidylinositol 3-kinase (PI3K; 1:1,000, #4257, Cell signaling), p-PI3K (1:1,000, #4228, Cell signaling), protein kinase B (Akt; 1:1,000, #9272, Cell signaling), p-Akt (1:1,000, #4060, Cell signaling), p-ERK (1:2,000, #9101, Cell signaling), glycogen synthase kinase-3β (GSK-3β; 1:2,000, #12456, Cell signaling), p-GSK-3β (1:2,000, #5558, Cell signaling), cleaved caspase 3 (1:500, #9661, Cell signaling), and bcl2 associated x protein (Bax; 1:1,000, AB7977, Abcam). Histological quantification was performed by an investigator blinded to group allocation.

### 2.11 Cell culture and treatment

HT22 mouse hippocampal cells were maintained in DMEM containing 10% heat-inactivated FBS and 1% penicillin/streptomycin at 37 °C in 5% CO_2_. Cells were seeded at 4 × 10^3^ cells/well and treated with AR at 3–300 μg/mL with or without AβO. For cell viability analysis, AR was applied 1 h before AβO exposure, and viability was measured after 23 h using the MTT assay (Lee et al., 2021). For immunofluorescence staining, cells were fixed, permeabilized, and incubated with an anti-microtubule-associated protein 2 antibody (1:1,000, SC-74421, Santa Cruz) overnight at 4 °C. After secondary antibody incubation and Hoechst nuclear staining, fluorescence signals were observed using a BX51 fluorescence microscope (Olympus, Tokyo, Japan). For Western blot analysis, cells were seeded in 12-well plates at 3 × 10^5^ cells/well and pretreated with AR at 30 or 300 μg/mL for 1 h before exposure to 5 μM AβO for 3 h. Protein expression was analyzed as described in Section 2.10.

The WA09 hESC line was obtained from WiCell and cultured on Laminin-511-coated plates in Essential 8 medium. To generate hippocampal neurons, hESCs were dissociated with Accutase and replated onto Geltrex-coated plates at 1 × 10^5^ cells/cm^2^ with 10 μM Y-27632. At approximately 70% confluency, cells were exposed for 5 days to neural induction medium containing N2, B27 without vitamin A, LDN193189, SB431542, IWP4, and cyclopamine. The resulting hippocampal neural progenitor cells were further differentiated for 10 days with CHIR99021, NGF, BDNF, ascorbic acid, and dibutyryl-cAMP, and then matured for another 10 days in the same medium without CHIR99021. Cells at passages 20–40 were used for differentiation. Biweekly *mycoplasma* testing confirmed that all cultures were negative.

### 2.12 Statistical analysis

All data are presented as the mean ± standard error of the mean (SEM). Statistical analyses were performed using GraphPad Prism version 8.0 (GraphPad Software, San Diego, CA, United States). Differences among groups were analyzed using one-way analysis of variance (ANOVA), followed by Dunnett’s multiple-comparisons test. Omnibus ANOVA results are reported as F values with the corresponding numerator and denominator degrees of freedom and p-values. Comparisons between individual groups were evaluated using Dunnett-adjusted p-values, with the relevant comparison group indicated in each figure legend. A p-value <0.05 was considered statistically significant.

## 3 Results

### 3.1 HPLC-UV analysis for AR

**FIGURE 1:**
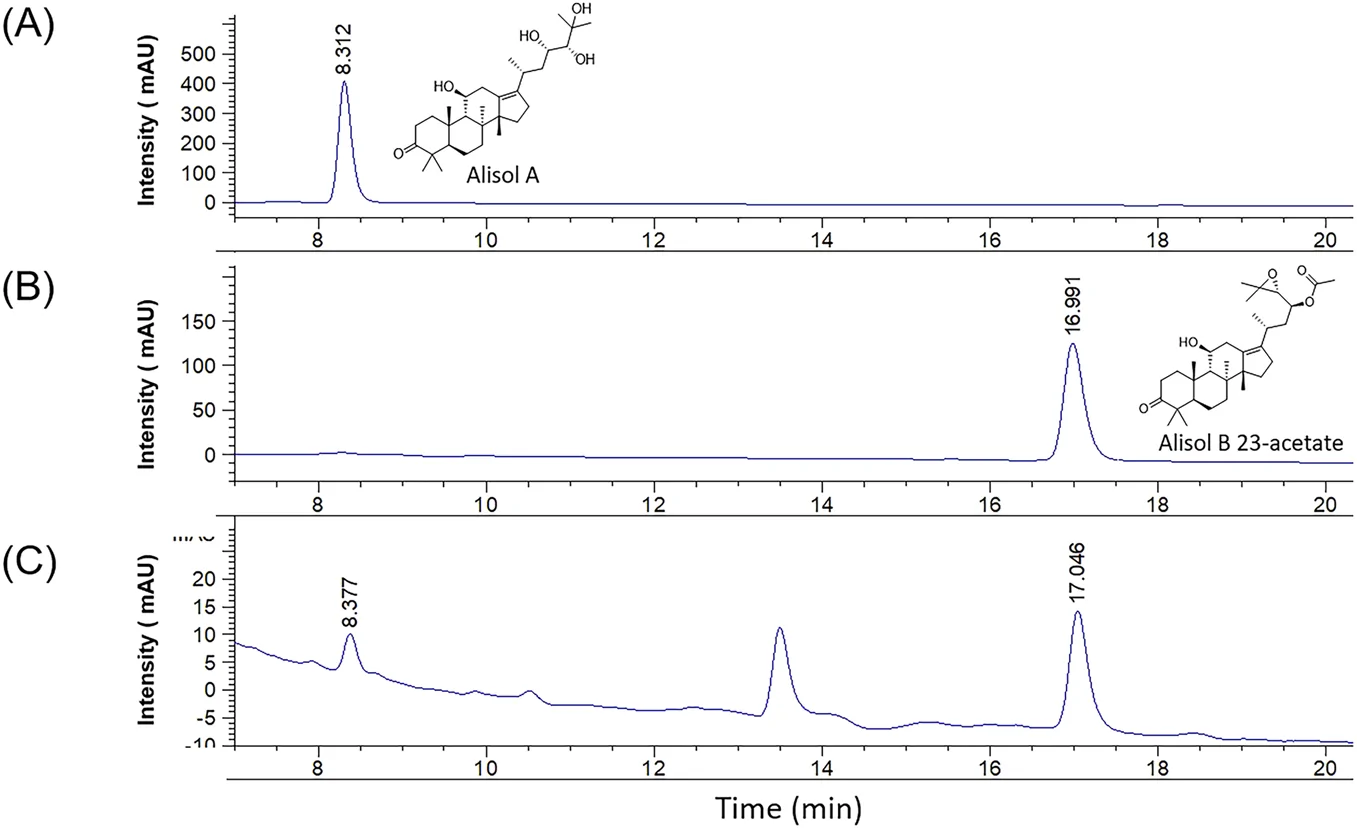
Standardization of AR. Alisol A standard solution [**(A)**; RT 8.312 min], alisol B 23-acetate standard solution [**(B)**; RT 16.991 min], and HPLC-UV chromatogram of AR extract **(C)** at 210 nm. Abbreviations: AR, Alismatis Rhizoma; RT, retention time; HPLC-UV, High-performance liquid chromatography with ultraviolet detection.

To standardize AR, we quantified AA and AB by matching their retention times with those of standard compounds. In AR samples, AA (Figure 1A) and AB (Figure 1B) were detected with retention times of 8.377 and 17.046 min (Figure 1C). For quantification, calibration curves were established using serially diluted standard solutions (3.91–250 μg/mL). The regression equations were Y = 9.303X – 22.056 for AA and Y = 9.614X + 13.861 for AB. The content of AA and AB in AR were determined to be 0.235 ± 0.010 and 0.504 ± 0.031 mg/g, respectively.

### 3.2 AR prevented memory deficits in AβO-injected mice

**FIGURE 2:**
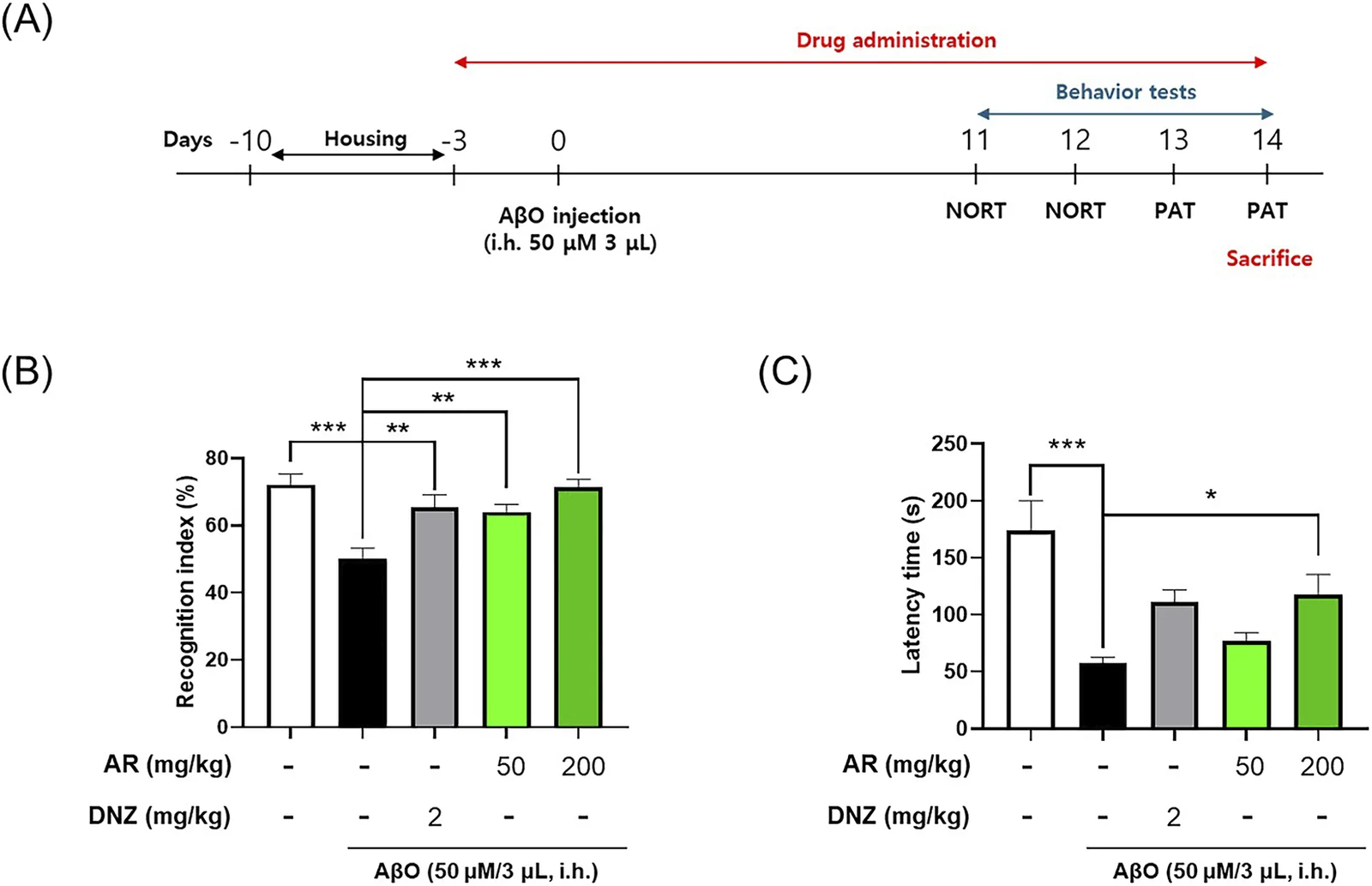
Experimental scheme and effects of AR on memory impairment in the AβO-injected mice. Timeline of drug administration and behavioral testing **(A)**. The NORT **(B)** and PAT **(C)** were performed to evaluate the effect of AR on memory function by measuring the recognition index **(B)** and latency time in the bright compartment **(C)**. Data were analyzed using ANOVA, followed by Dunnett’s *post hoc* test. ***p < 0.001, **p < 0.01 and *p < 0.05 vs. the AβO group. Abbreviations: AR, Alismatis Rhizoma; AβO, amyloid-β oligomer; NORT, novel object recognition test; PAT, passive avoidance test; ANOVA, one-way analysis of variance.

We first assessed the effects of AR on AβO-induced memory impairment *in vivo*. The experimental schedule is shown in Figure 2A. Mice receiving an intrahippocampal AβO injection were orally administered AR at 50 or 200 mg/kg, with DNZ serving as the positive control. AβO injection markedly reduced the recognition index compared with the sham group, whereas AR treatment at 50 or 200 mg/kg increased the recognition index to levels comparable to those observed with DNZ (Figure 2B). In the PAT, AβO injection significantly shortened the step-through latency, while 200 mg/kg AR prolonged it (Figure 2C). The omnibus ANOVA results were as follows: Figure 2B: F(4,54) = 9.406, p < 0.0001; Figure 2C: F(4,61) = 7.101, p < 0.0001.

### 3.3 AR prevented neuronal death and synaptic damage in AβO-injected mice

**FIGURE 3:**
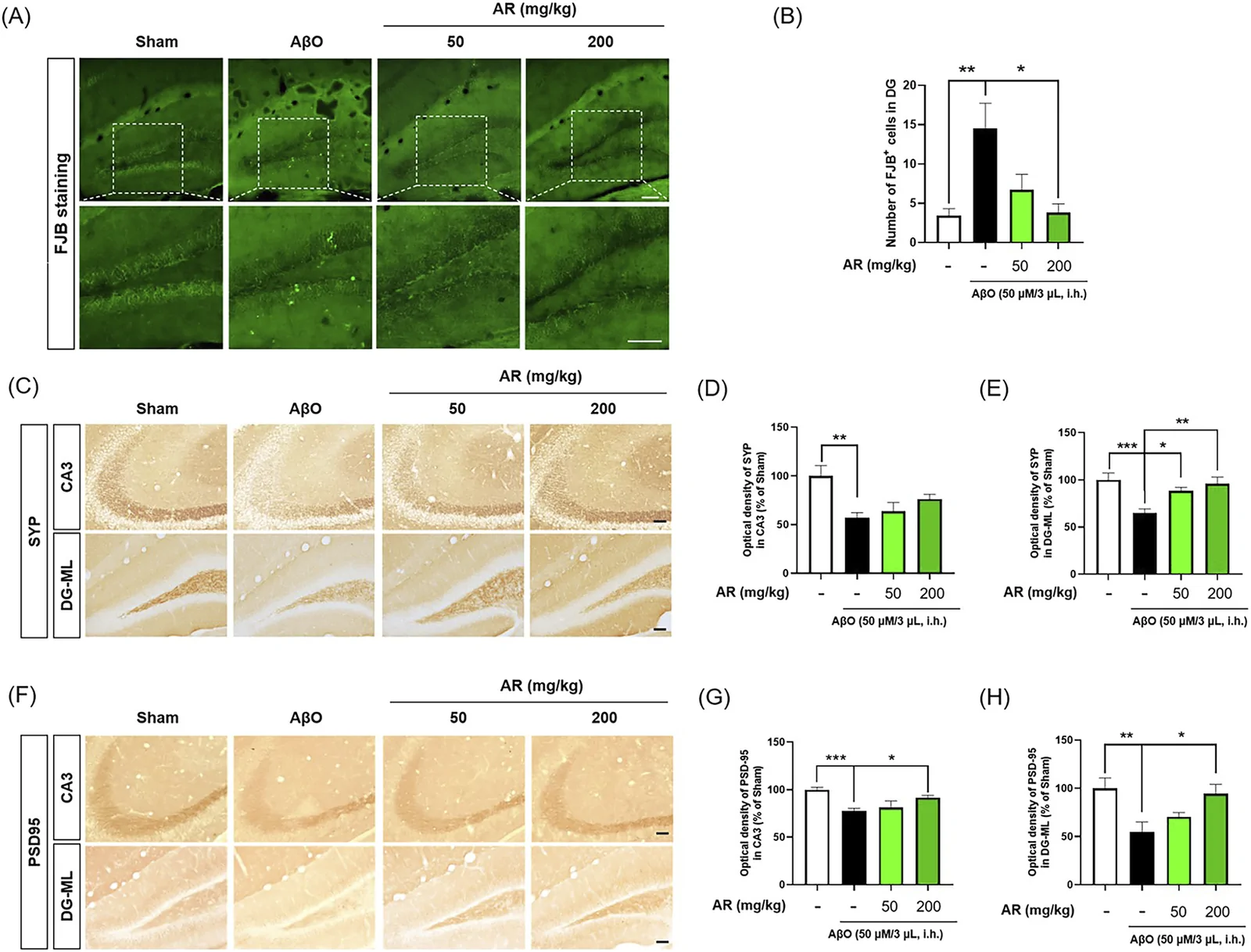
Effects of AR on neuronal cell death and synaptic loss in the AβO-injected mice. Representative images **(A)** and quantification **(B)** of FJB-positive cells in the dentate gyrus are shown. Representative immunofluorescence images of SYP and PSD-95 in the CA3 and DG-ML are shown. SYP **(C–E)** and PSD-95 **(F–H)** immunoreactivities were quantified as optical density in both hippocampal regions. The data were analyzed using ANOVA, followed by Dunnett’s *post hoc* test. ***p < 0.001, **p < 0.01 and *p < 0.05 vs. the AβO group. Scale bar = 100 μm. Abbreviations: AR, Alismatis Rhizoma; AβO, amyloid-β oligomer; SYP, synaptophysin; PSD-95, postsynaptic density protein 95; CA3, cornu ammonis 3; DG, dentate gyrus molecular layer; ANOVA, one-way analysis of variance.

To investigate whether AR ameliorates AβO-induced neuronal cell death, we performed FJB-staining to confirm the neuroprotective effect of AR in the hippocampus. AβO injection significantly increased the number of FJB-positive cells in the hippocampus, whereas AR treatment at 200 mg/kg markedly reduced this increase (Figures 3A,B).

We next examined the synaptic proteins SYP (Figures 3C–E) and PSD-95 (Figures 3F–H) in the CA3 stratum lucidum and DG-ML. AβO injection reduced SYP and PSD-95 immunoreactivity in both regions, while AR treatment at 200 mg/kg attenuated these reductions. The omnibus ANOVA results were as follows: Figure 3B: F(3,22) = 5.780, p = 0.0045; Figure 3D: F(3,22) = 6.204, p = 0.0032. Figure 3E: F(3,23) = 8.002, p = 0.0008; Figure 3G: F(3,23) = 7.129, p = 0.0015; Figure 3H: F(3,23) = 5.208, p = 0.0068.

### 3.4 AR enhanced BDNF-related signaling in AβO-injected mice

**FIGURE 4:**
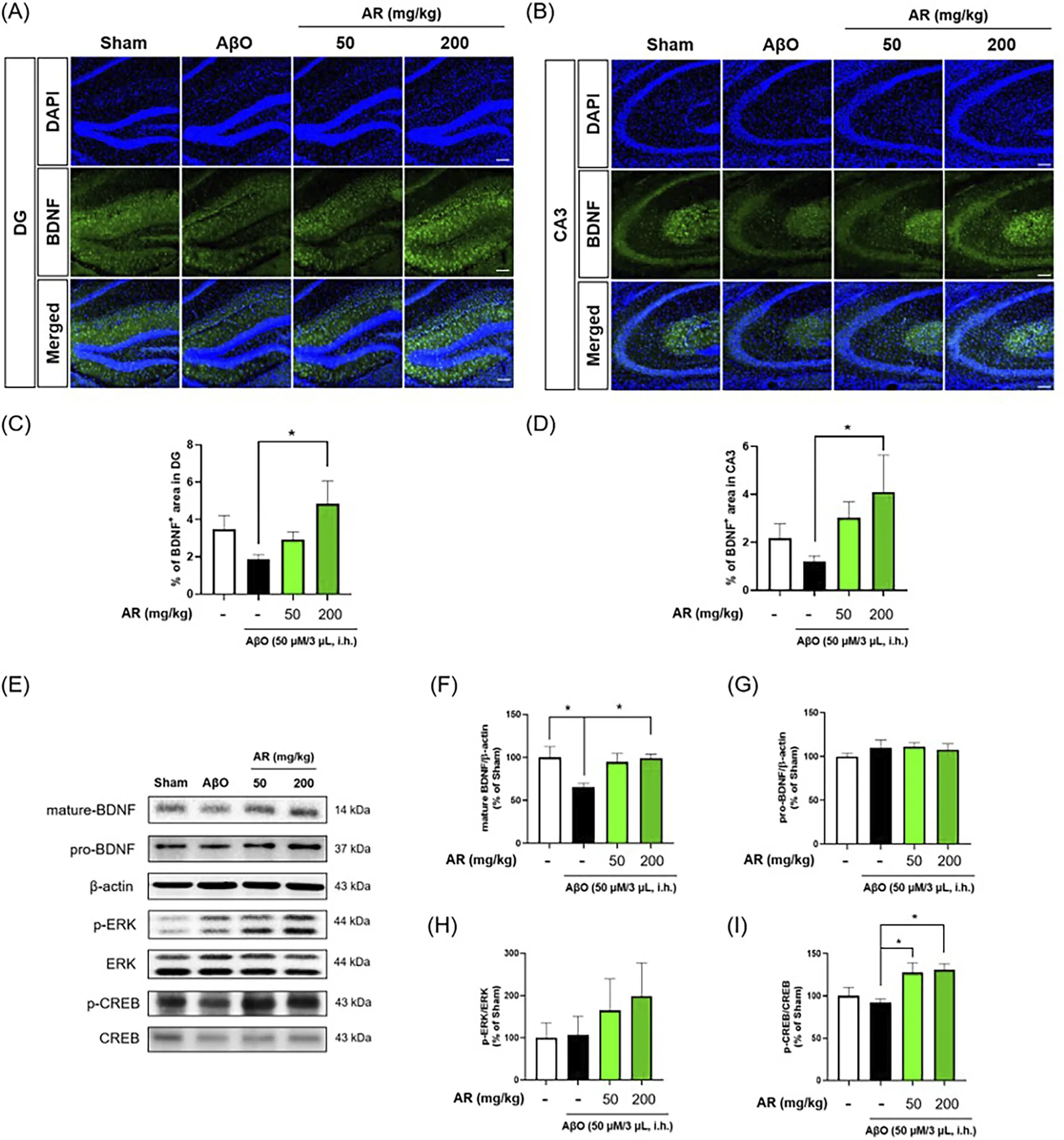
Effects of AR on BDNF-related signaling in the hippocampus of AβO-injected mice. Representative photomicrographs **(A, B)** and quantification **(C, D)** of BDNF- (green) and DAPI-positive areas (blue) in the hippocampal DG and CA3 region are shown. Representative Western blot bands are shown in **(E)**, and quantification of mature BDNF and pro-BDNF protein levels normalized to β-actin is shown in **(F)** and **(G)**. Quantification of phosphorylated ERK and CREB normalized to total ERK and CREB levels is shown in **(H)** and **(I)**. The data were analyzed using ANOVA, followed by Dunnett’s *post hoc* test. **p < 0.01 and *p < 0.05 vs. the AβO group. Scale bar = 100 μm. Abbreviations: AR, Alismatis Rhizoma; AβO, amyloid-β oligomer; BDNF, brain-derived neurotrophic factor; DAPI, 4′,6-diamidino-2-phenylindole; DG, dentate gyrus molecular layer; CA3, cornu ammonis 3; ERK, extracellular signal-regulated kinase; CREB, cAMP response element-binding protein; ANOVA, one-way analysis of variance.

To assess whether AR upregulates BDNF expression, we performed immunohistochemical analysis and quantified the BDNF-positive area in the DG and CA3 regions in hippocampus. AβO injection tended to reduce the BDNF-positive area in both regions compared with the sham group, although these differences did not reach statistical significance. AR treatment at 200 mg/kg significantly increased BDNF immunoreactivity in the DG (Figures 4A,C) and CA3 (Figures 4B,D) compared with the AβO group.

To distinguish between pro-BDNF and mature BDNF based on molecular weight, we performed Western blot analysis (Figure 4E). Pro-BDNF expression showed no significant variation among the groups (Figure 4G). In contrast, mature BDNF expression significantly decreased upon AβO injection, whereas AR treatment at 200 mg/kg markedly preserved its expression (Figure 4F). Moreover, we examined whether AR affected the phosphorylation of ERK and CREB, which are associated with BDNF expression. AR treatment at 200 mg/kg increased the p-ERK/ERK and p-CREB/CREB ratios (Figures 4H,I). The omnibus ANOVA results were as follows: Figure 4C: F(3,23) = 3.155, p = 0.0442; Figure 4D: F(3,23) = 2.338, p = 0.1001; Figure 4F: F(3,20) = 3.441, p = 0.0364; Figure 4G: F(3,20) = 0.6399, p = 0.5982; Figure 4H: F(3,20) = 0.5995, p = 0.6228; Figure 4I: F(3,20) = 5.018, p = 0.0094.

### 3.5 AR stimulated neuronal cell proliferation and differentiation in AβO-injected mice

**FIGURE 5:**
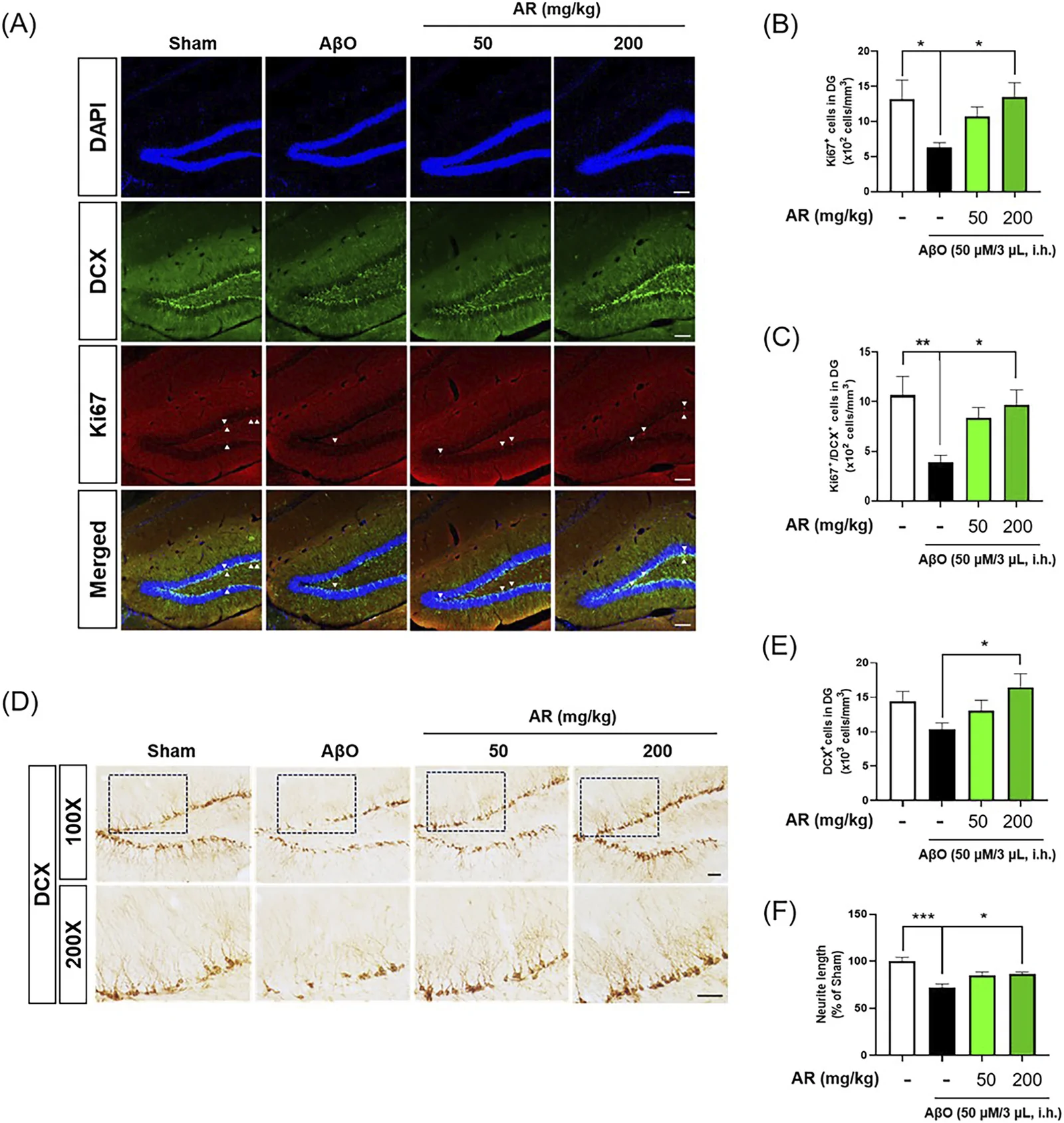
Effects of AR on neuronal cell proliferation and differentiation in the hippocampus of AβO-injected mice. Immunofluorescence images of DCX- (green), Ki67- (red), and DAPI-stained areas (blue) in the hippocampus are shown **(A)**, with quantification of Ki67-positive cells and DCX/Ki67 double-positive cells **(B, C)** performed using ImageJ. Representative images of DCX immunostaining **(D)** and corresponding quantification **(E, F)** are shown. The data were analyzed using ANOVA, followed by Dunnett’s *post hoc* test. ***p < 0.001, **p < 0.01, and *p < 0.05 vs. the AβO group. Scale bar = 100 μm. Abbreviations: AR, Alismatis Rhizoma; AβO, amyloid-β oligomer; DCX, doublecortin; DAPI, 4′,6-diamidino-2-phenylindole; ANOVA, one-way analysis of variance.

In the brain, BDNF enhanced proliferation and differentiation of neuronal cells (Lozano-Urena and Frade, 2023). To assess the effect of AR on cell proliferation, we measured the number of Ki67-positive cells in the hippocampus. The AβO group had a significantly lower number of Ki67-positive cells than the Sham group, but AR treatment at 200 mg/kg markedly preserved their numbers (Figures 5A,B). Moreover, to investigate neuronal cells actively undergoing both proliferation and differentiation, we performed co-staining for Ki67 and DCX in the hippocampus. The number of cells co-stained with Ki67 and DCX was lower in the AβO group than in the sham group; however, AR treatment significantly increased the number of co-stained cells in the hippocampus (Figures 5A,C).

To investigate whether AR stimulates neuronal cell differentiation, we performed immunohistochemical analysis using an antibody against DCX, which is commonly used as a marker for neuronal precursor cells and immature neurons. We observed that neurite outgrowth and the number of DCX-positive cells were lower in the AβO group than in the sham group; however, AR administration at 200 mg/kg significantly increased both neurite outgrowth and the number of DCX-positive cells (Figures 5D–F). The omnibus ANOVA results were as follows: Figure 5B: F(3,23) = 3.623, p = 0.0282; Figure 5C: F(3,23) = 5.574, p = 0.0050; Figure 5E: F(3,23) = 3.255, p = 0.0401; Figure 5F: F(3,23) = 10.04, p = 0.0002.

### 3.6 AR modulated PI3K/Akt/GSK-3β signaling and attenuated apoptotic signaling in AβO-injected mice

**FIGURE 6:**
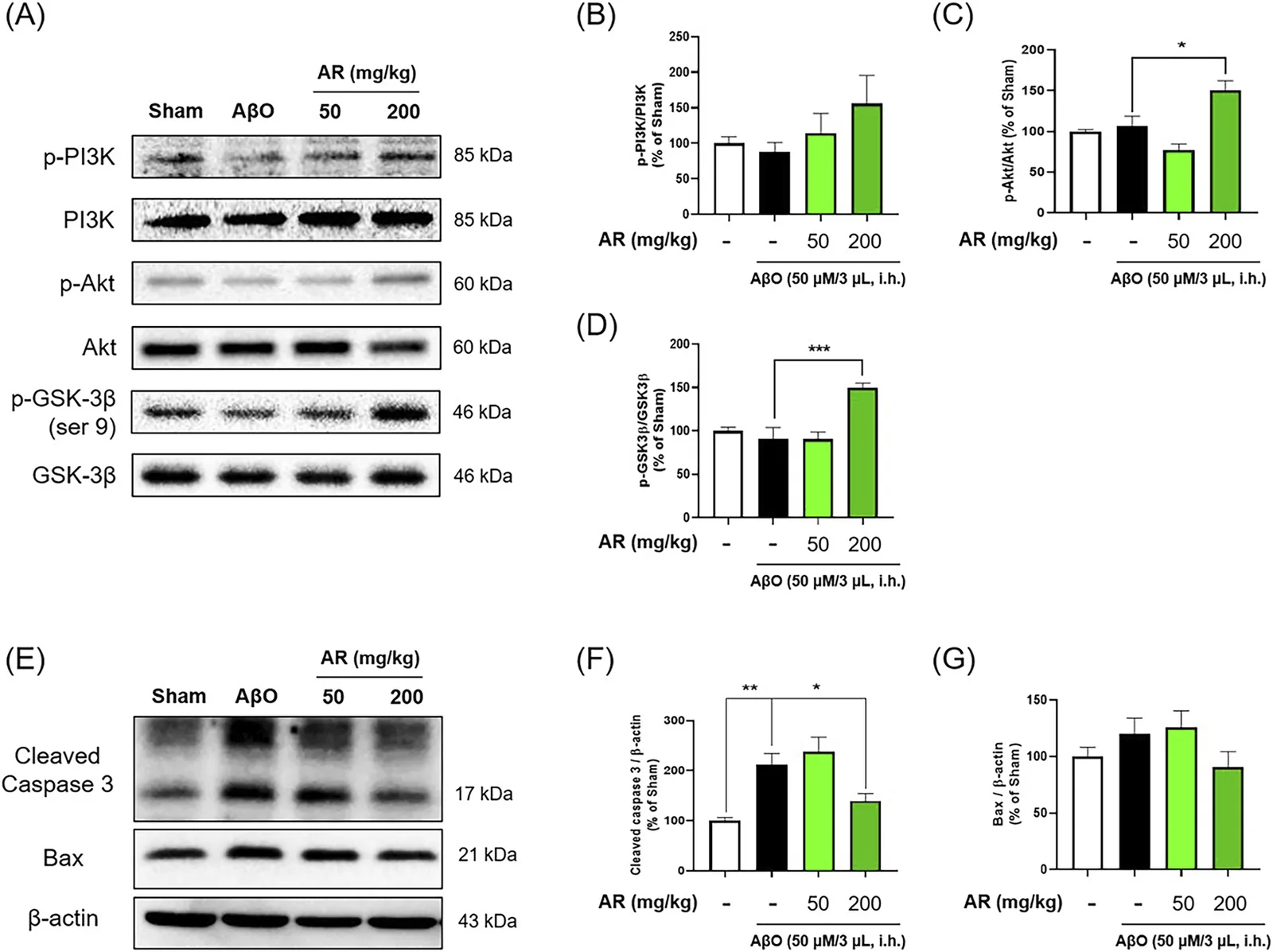
Effects of AR on PI3K/Akt/GSK-3β signaling and apoptosis-related proteins in AβO-injected mice. Representative Western blot bands are shown in **(A)**, and quantification of phosphorylated PI3K, Akt, and GSK-3β normalized to total PI3K, Akt, and GSK-3β levels is shown in **(B**–**D)**. Representative Western blot bands for Cleaved caspase-3 and Bax are shown in **(E)**, and their expression levels were normalized to β-actin and quantified in **(F)** and **(G)**, respectively. The data were analyzed using ANOVA, followed by Dunnett’s *post hoc* test. ***p < 0.001 and *p < 0.05 vs. the AβO group. Abbreviations: AR, Alismatis Rhizoma; AβO, amyloid-β oligomer; PI3K, phosphoinositide 3-kinase; Akt, protein kinase B; GSK-3β, glycogen synthase kinase-3 beta; Bax. bcl2 associated x protein; ANOVA, one-way analysis of variance.

PI3K/Akt/GSK-3β signaling regulates neuronal survival and apoptosis (Kitagishi et al., 2014). We therefore examined the phosphorylation of PI3K, Akt, and GSK-3β, together with the apoptosis-related proteins cleaved caspase-3 and Bax, in the hippocampus. The p-PI3K/PI3K, p-Akt/Akt, and p-GSK-3β/GSK-3β ratios did not significantly differ between the sham and AβO groups. However, AR treatment at 200 mg/kg increased all three phosphorylation ratios compared with the AβO group (Figures 6A–D). AβO injection also increased cleaved caspase-3 expression, whereas AR 200 mg/kg significantly attenuated this increase (Figures 6E,F). Bax expression showed a decreasing trend following AR treatment, although the difference did not reach statistical significance (Figures 6E,G). The omnibus ANOVA results were as follows: Figure 6B: F(3,20) = 1.344, p = 0.2883; Figure 6C: F(3,20) = 10.31, p = 0.0003; Figure 6D: F(3,20) = 11.52, p = 0.0001; Figure 6F: F(3,20) = 10.07, p = 0.0003; Figure 6G: F(3,20) = 1.659, p = 0.2078.

### 3.7 AR attenuated AβO-induced neurotoxicity in the hippocampal cells

**FIGURE 7:**
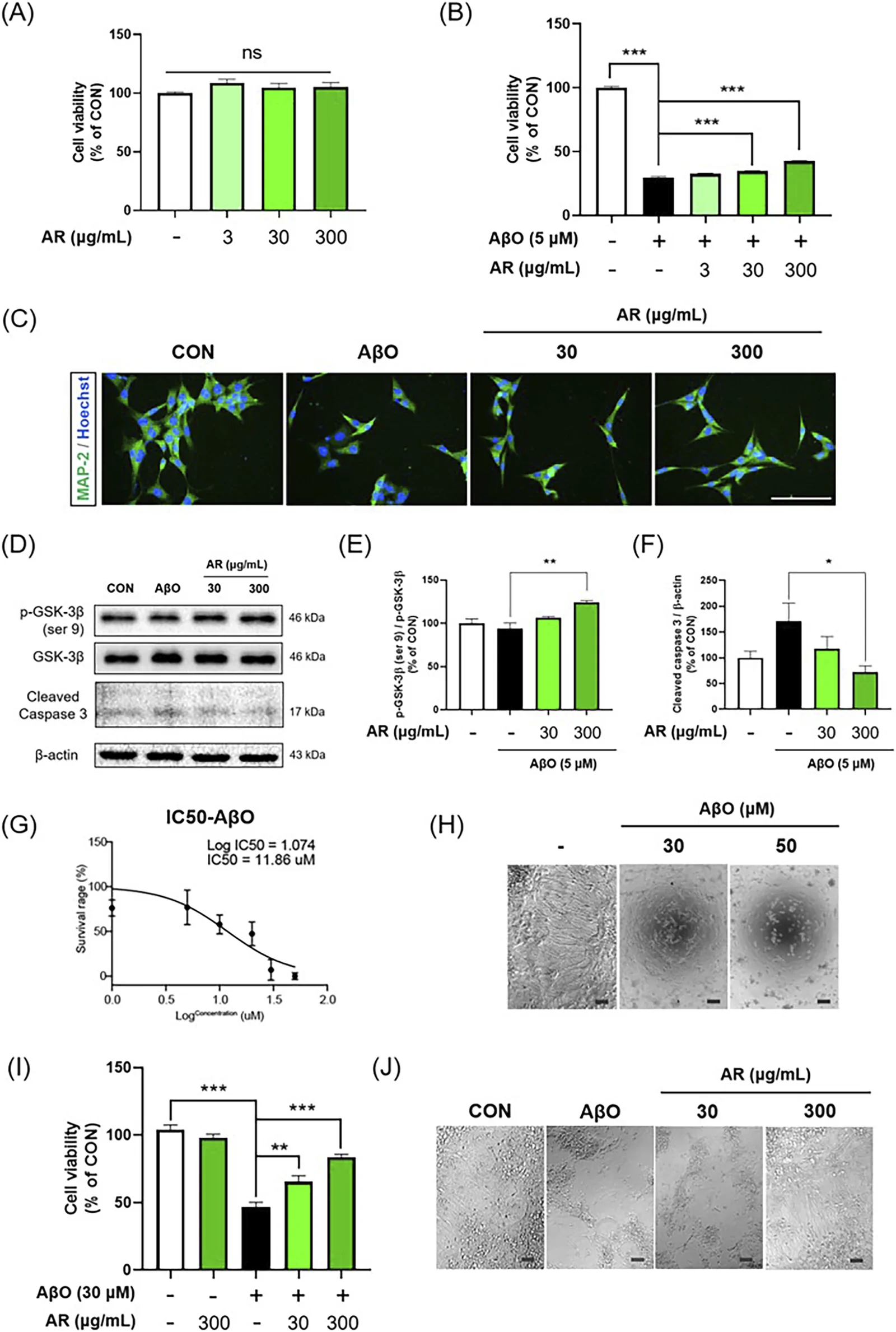
Effects of AR on AβO-induced neuronal toxicity in the hippocampal neuronal models. HT22 cells were treated with AR at various doses for 24 h **(A)** or pre-treated with AR for 1 h followed by treatment with 50 μM AβO for 23 h **(B)**. Cell viability was measured using the MTT assay. Representative images of HT22 cells are shown **(C)**. For Western blot analysis, HT22 cells were pretreated with 30 or 300 μg/mL AR for 1 h and then exposed to 5 μM AβO for 3 h. Representative bands are shown in **(D)**. Cleaved caspase-3 levels normalized to β-actin **(E)** and the p-GSK-3β (Ser9)/GSK-3β ratio **(F)** were quantified. The IC_50_ was calculated based on neuronal viability measured using the MTT assay **(G)**. Morphological assessment of hESC-derived hippocampal neurons after 24 h treatment with 0, 30, or 50 μM AβO **(H)**. Neurons were pretreated with 30 or 300 μg/mL AR for 24 h, followed by 30 μM AβO treatment for an additional 24 h. Neuronal viability was quantified **(I)**, and representative immunofluorescence images showing axodendritic structure are presented **(J)**. The data were analyzed using ANOVA, followed by Dunnett’s *post hoc* test. ***p < 0.001 and **p < 0.01 vs. the AβO-only treated group. Scale bar = 100 μm. Abbreviations: AR, Alismatis Rhizoma; AβO, amyloid-β oligomer; MTT, 3-(4,5-dimethylthiazol-2-yl)-2,5-diphenyltetrazolium bromide; GSK-3β, glycogen synthase kinase-3 beta; IC_50_, half-maximal inhibitory concentration; hESC, human embryonic stem cells; ANOVA, one-way analysis of variance.

To confirm the protective effects of AR in hippocampal neuronal models, we examined both murine HT22 cells and hESC-derived hippocampal neurons. In HT22 cells, AR alone did not affect cell viability, whereas AβO markedly reduced it. AR pretreatment significantly attenuated this reduction (Figures 7A,B), and representative images qualitatively reflected the viability results (Figure 7C). AR also reduced cleaved caspase-3 levels and tended to increase the p-GSK-3β (Ser9)/GSK-3β ratio (Figures 7D–F).

We next evaluated AR in hESC-derived hippocampal neurons differentiated for 30 days. AβO exposure for 24 h reduced neuronal viability in a concentration-dependent manner, yielding an IC_50_ of 11.86 μM (Figure 7G). AβO at 30 μM caused more than 70% neuronal death, while 50 μM resulted in near-complete loss of viability (Figure 7H). Pretreatment with 30 or 300 μg/mL AR significantly increased neuronal survival following AβO exposure. Pretreatment with 30 or 300 μg/mL AR significantly attenuated the AβO-induced reduction in neuronal viability (Figure 7I). Pretreatment with 30 or 300 μg/mL AR significantly attenuated the AβO-induced reduction in neuronal viability, and representative images qualitatively reflected these changes (Figures 7I,J). The omnibus ANOVA results were as follows: Figure 7A: F(3,22) = 1.426, p = 0.2619; Figure 7B: F(4,27) = 1,585, p < 0.0001; Figure 7E: F(3,12) = 8.361, p = 0.0029; Figure 7F: F(3,12) = 3.450, p = 0.0515; Figure 7I: F(4,10) = 50.34, p < 0.0001.

## 4 Discussion

In this study, we used complementary cellular and animal models to investigate the protective effects of AR against acute AβO-induced hippocampal injury. Synaptic dysfunction occurs early in AD and is closely associated with cognitive decline (Taddei and Duff, 2025). Soluble AβO accumulates at synaptic sites and disrupts calcium homeostasis, mitochondrial function, and long-term potentiation, thereby contributing to synaptic loss, neuronal injury, and memory impairment (Figueiredo et al., 2013; Tu et al., 2014; Pickett et al., 2016). AR preserved pre- and postsynaptic protein immunoreactivity in parallel with reduced neuronal degeneration and memory deficits. These findings suggest that maintenance of synaptic integrity may contribute to the protective effects of AR under acute AβO-induced conditions.

The PI3K/Akt signaling pathway promotes the upregulation of BDNF, which is essential for neuronal growth, survival, and synaptic plasticity (Srivastava et al., 2018). Activation of this pathway has been reported to enhance the synthesis of synaptic proteins and the development of the cytoskeleton, both of which are essential for the enhancement of synaptic plasticity (Guo et al., 2024). Moreover, Akt acts as a kinase that phosphorylates and thereby inactivates GSK-3β, an inhibitory regulator of synaptic integrity and plasticity (Lauretti et al., 2020). Activation of GSK-3β has been observed in both the brain and plasma of patients with AD (Zhou et al., 2022). Cuesto et al. reported that the activation of GSK-3β in a *Drosophila* model induced synaptic loss, whereas its genetic suppression increased synapse number (Cuesto et al., 2015). They also reported that the pharmacological inhibition of GSK-3β in rat hippocampal neurons enhanced synaptic density and synapsin. Moreover, GSK-3β activation has also been implicated in the disruption of hippocampal long-term potentiation, a process critical for learning and memory functions (Hooper et al., 2007). In addition, activated GSK-3β suppresses CREB phosphorylation, thereby limiting CREB-dependent transcriptional programs involved in synaptic plasticity and BDNF expression (Grimes and Jope, 2001; Amidfar et al., 2020). Importantly, dysregulation of GSK-3β signaling has also been linked to neuronal survival pathways, including caspase-dependent apoptosis. Increased GSK-3β activity has been associated with elevated cleaved caspase-3 levels under AD-related conditions, suggesting a potential link between this pathway and neuronal cell death (Hu et al., 2009). Consistently, enhanced caspase-3 activation has been observed in AD and is associated with progressive neuronal loss and cognitive impairment (Wojcik et al., 2024). Consistent with these observations, AR treatment was associated with reduced cleaved caspase-3 levels in the hippocampus. In parallel, AR increased the phosphorylation of PI3K, Akt, and GSK-3β, indicating that these changes may be associated with reduced apoptosis-related signaling under AβO-induced neurotoxic conditions.

BDNF plays a pivotal role in neuronal survival, synaptic plasticity, and cognitive function (Bathina and Das, 2015). The ERK/CREB signaling pathway is closely associated with the regulation of BDNF expression, as ERK activation may promote CREB phosphorylation, which in turn regulates BDNF expression (Ying et al., 2002). BDNF exists in precursor and mature forms, each of which exerts different biological actions. These two forms exhibit distinct and often opposing biological effects (Miranda et al., 2019). Mature BDNF preferentially binds to the tropomyosin receptor kinase B receptor, promoting neuronal survival, synaptic strengthening, neurotransmitter release, ion channel modulation, and neuronal excitability (Choo et al., 2017). In contrast, pro-BDNF primarily interacts with the p75 neurotrophin receptor, which can induce synaptic retraction and apoptosis (Wang et al., 2024). In AD, a pathological imbalance between pro-BDNF and mature BDNF levels has been reported, with a relative reduction in mature BDNF levels contributing to synaptic dysfunction and cognitive decline (Li et al., 2022). In the present study, AR increased mature BDNF levels without altering pro-BDNF and enhanced ERK and CREB phosphorylation in the hippocampus. These coordinated changes suggest that AR may support BDNF-related neuronal survival and synaptic plasticity in association with ERK/CREB signaling.

Neuronal cell proliferation and differentiation are critical processes for maintaining brain homeostasis and facilitating neural repair, as these processes involve the generation of new neurons and maturation of neuronal precursor cells, essential for neuroplasticity and memory functions (Jastrzebski et al., 2024). Studies have reported significant reductions in cell proliferation and differentiation, particularly in the hippocampus, in patients with AD (Zhou et al., 2023). In particular, Lee and colleagues showed that AβO suppresses the proliferative and differentiation capacities of human neural stem cells through alterations in GSK-3β signaling (Lee et al., 2013). Moreover, a previous study has reported that newly generated, immature granule neurons in the adult hippocampus exhibit enhanced synaptic plasticity, including increased susceptibility to long-term potentiation (Schmidt-Hieber et al., 2004). Furthermore, Lods et al. suggested that immature neurons generated during learning are reactivated during memory retrieval and that they are essential for maintaining and updating remote memory, indicating that their generation contributes directly to memory formation and stabilization (Lods et al., 2021). On the basis of these reports, the upregulation of cell proliferation and differentiation is considered to enhance synaptic function, which may contribute to improved memory function. These effects suggest that AR-mediated increases in cell proliferation and differentiation could be involved in maintaining synaptic integrity and memory function.

Several limitations should be considered when interpreting the present findings. The relatively short AR administration period limited the assessment of long-term neurogenesis and whether it contributed to the observed memory-protective effects. In addition, acute AβO exposure does not fully recapitulate the chronic and progressive nature of AD, including sustained endogenous Aβ accumulation, tau pathology, and aging-related changes. Longer-term studies using progressive amyloid- and tau-based models, such as 5xFAD and PS19 mice, are therefore needed. Finally, although AR exerted protective effects in hESC-derived hippocampal neurons, findings from this human-derived *in vitro* model cannot be directly extrapolated to clinical efficacy in patients with AD. Thus, the present results should be regarded as basic preclinical evidence rather than direct evidence supporting the therapeutic application of AR in AD.

In conclusion, this study provides preliminary experimental support for exploring the neuroprotective application of AR beyond its traditional indications. AR attenuated acute AβO-induced hippocampal neuronal and synaptic injury and the associated memory impairment, accompanied by changes in signaling related to neuronal survival, apoptosis, and synaptic plasticity. These findings provide basic preclinical evidence for the preventive neuroprotective effects of AR against acute AβO-induced injury and support its further evaluation in chronic and progressive AD models.

## Data Availability

The original contributions presented in the study are included in the article/Supplementary Material, further inquiries can be directed to the corresponding author.
