# Supplementary material for: Alismatis Rhizoma prevents AβO-induced neuronal cell death and synaptic loss in Alzheimer’s disease models
Source: Front Pharmacol. 2026 Sep 4;17:1898356. doi: 10.3389/fphar.2026.1898356 (PMC13586005; doi:10.3389/fphar.2026.1898356)

✓ Protein ladder information

PageRuler™ Prestained Protein Ladder, 10 to 180 kDa (Cat No. 26616, Thermo fisher)

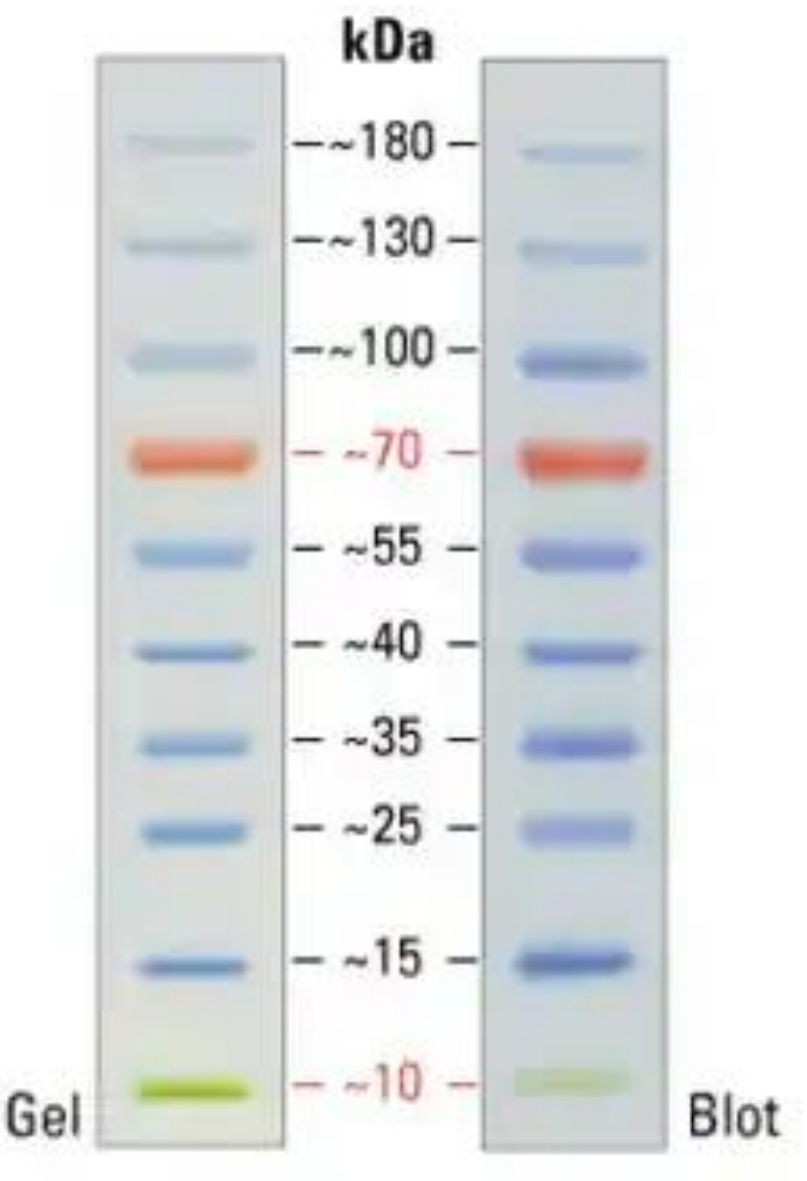

https://www.thermofisher.com/order/catalog/product/kr/ko/26616

✓ Antibody information

BDNF antibody (AB108319, Abcam)

HRP conjugated β-actin antibody (SC-47778HRP, Santa cruz)

Phospho-CREB antibody (#9198, Cell signaling)

CREB antibody (06-863, Millipore)

Phospho-ERK antibody (#9101, Cell signaling)

ERK antibody (SC-93, Santa cruz)

Phospho-PI3K antibody (#4228, Cell signaling)

PI3K antibody (#4257, Cell signaling)

Phospho-Akt antibody (#4060, Cell signaling)

Akt antibody (#9272, Cell signaling)

Phospho-GSK-3β antibody (#5558, Cell signaling)

GSK-3β antibody (#12456, Cell signaling)

Cleaved caspase 3 antibody (#9661, Cell signaling)

Bax antibody (AB7977, Abcam)

Blot images in Fig. 4

Code in blot images	
Sh (n=6)	Sham
Ab (n=6)	AβO
AR50 (n=6)	AβO+AR 50 mg/kg
AR200 (n=6)	AβO+AR 200 mg/kg

BDNF/β-actin
(no. 1~6 in each group)

highlight the protein bands (used in this manuscript with molecular weight details)

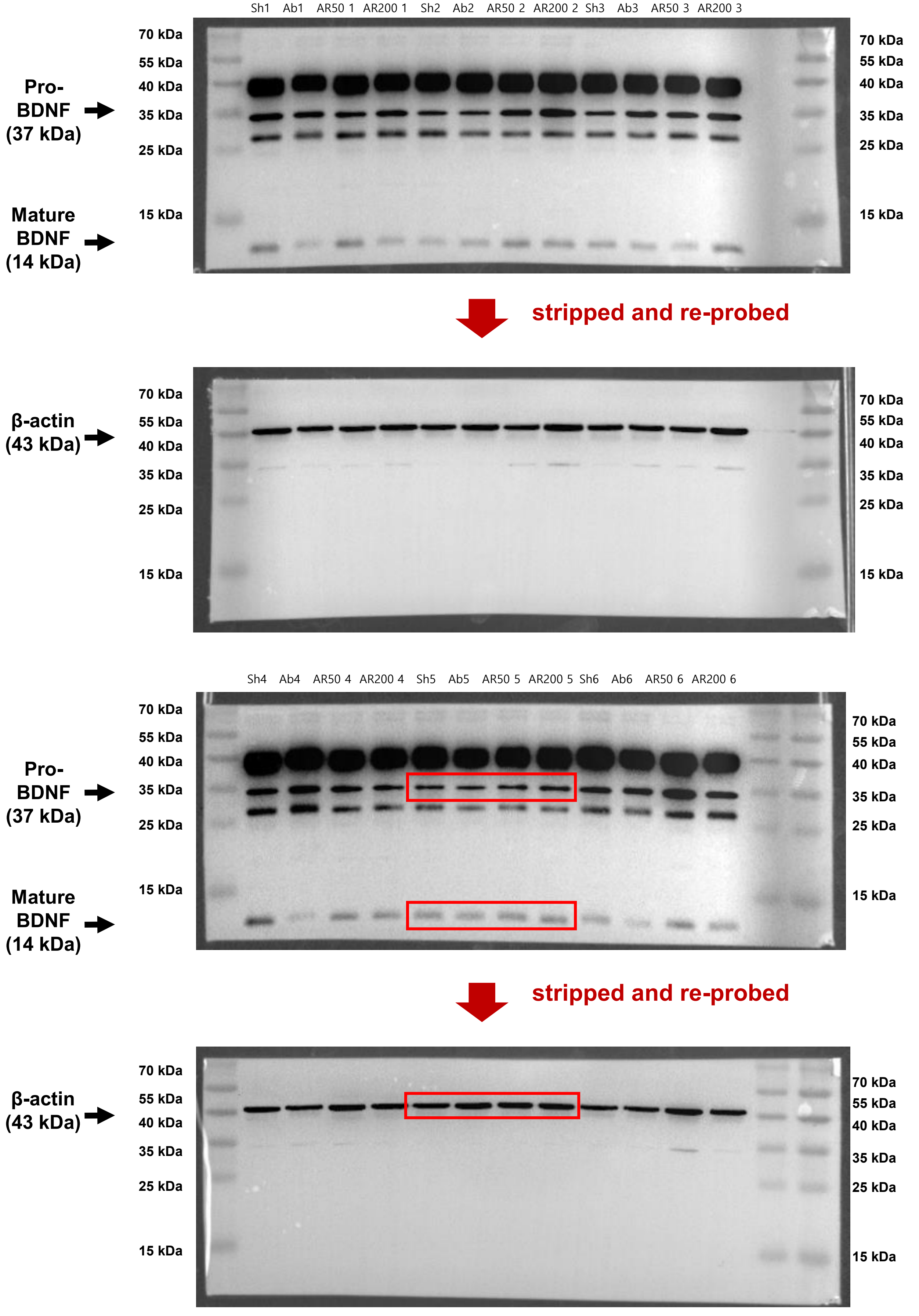

Blot images in Fig. 4

Code in blot images	
Sh (n=6)	Sham
Ab (n=6)	AβO
AR50 (n=6)	AβO+AR 50 mg/kg
AR200 (n=6)	AβO+AR 200 mg/kg

p-CREB/CREB
(no. 1~6 in each group)

highlight the protein bands (used in this manuscript with molecular weight details)

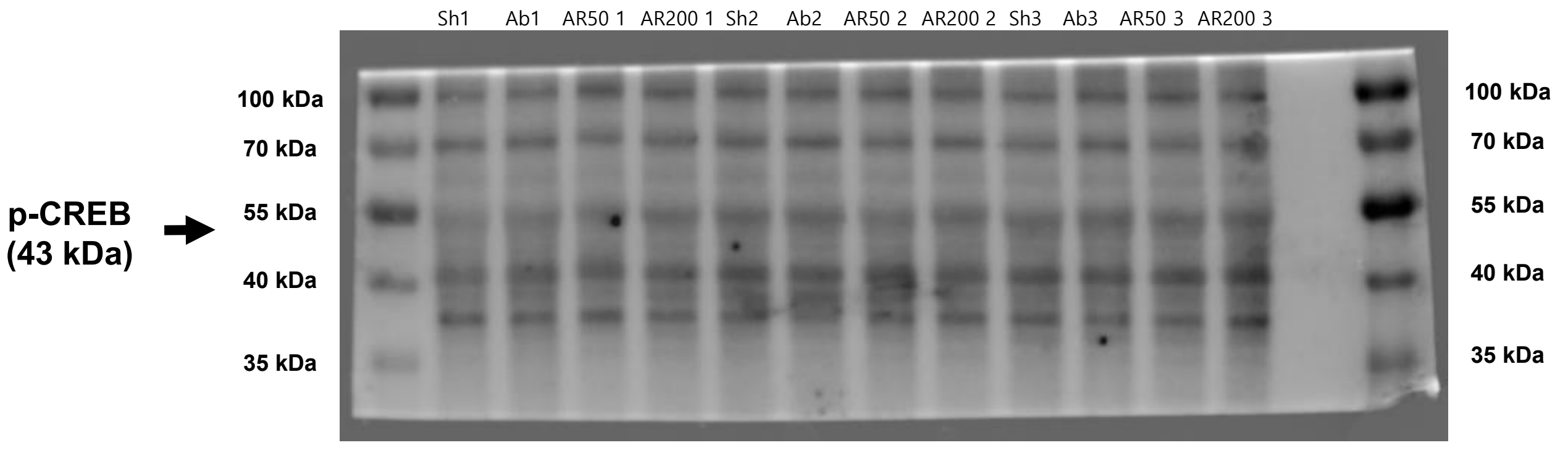

stripped and re-probed

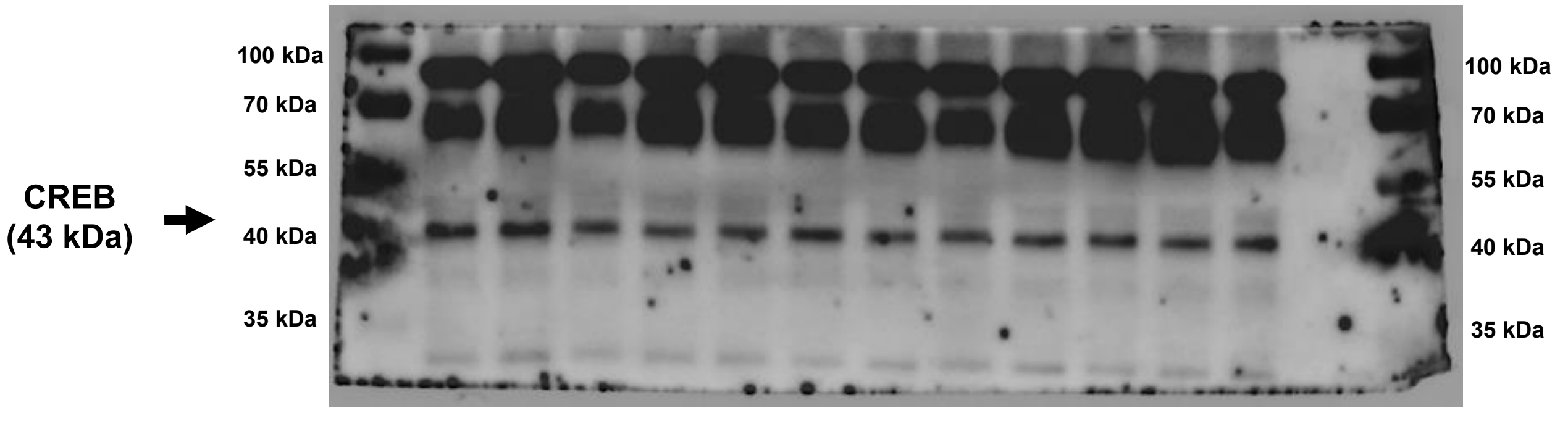

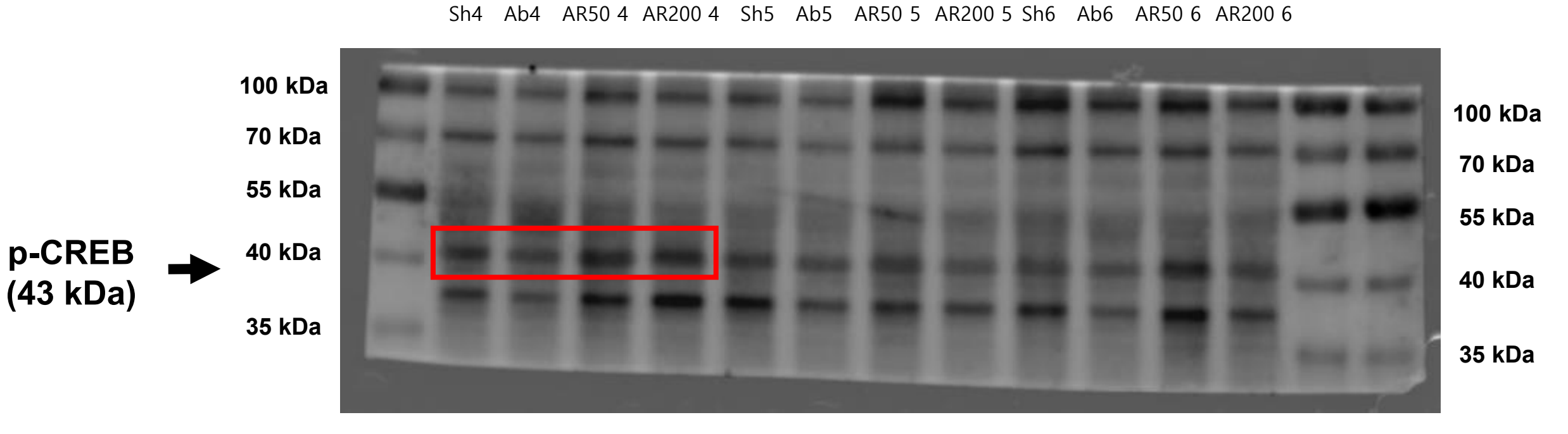

stripped and re-probed

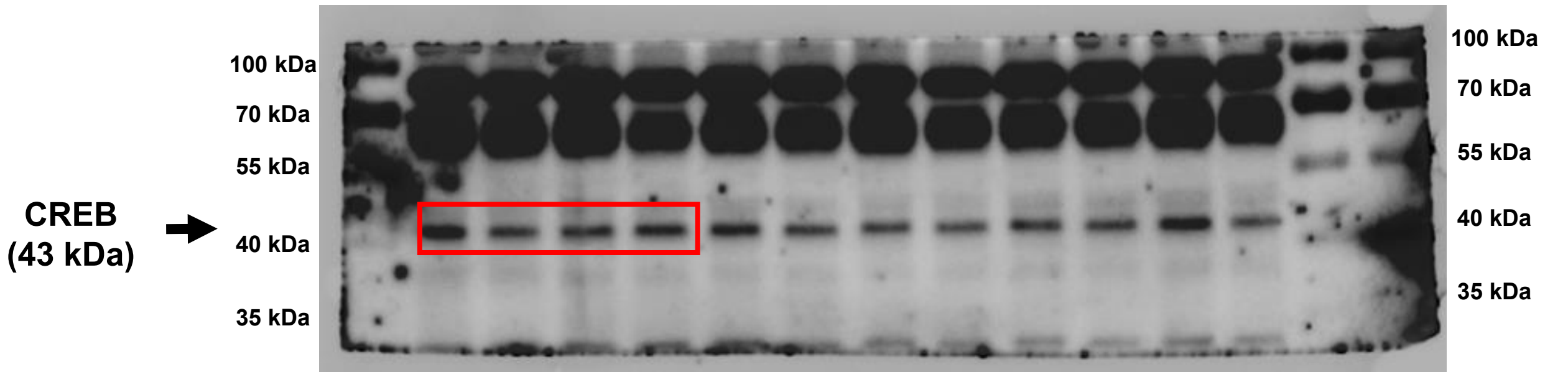

Blot images in Fig. 4

Code in blot images	
Sh (n=6)	Sham
Ab (n=6)	AβO
AR50 (n=6)	AβO+AR 50 mg/kg
AR200 (n=6)	AβO+AR 200 mg/kg

p-ERK/ERK
(no. 1~6 in each group)

highlight the protein bands (used in this manuscript with molecular weight details)

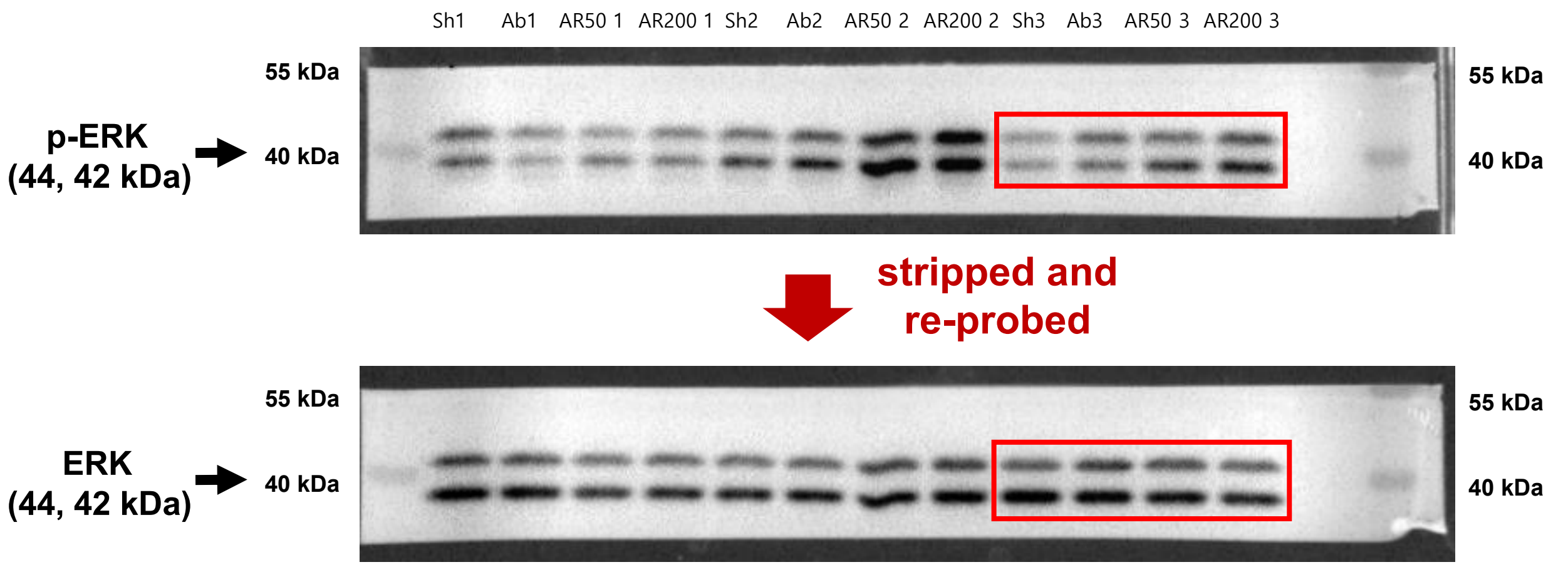

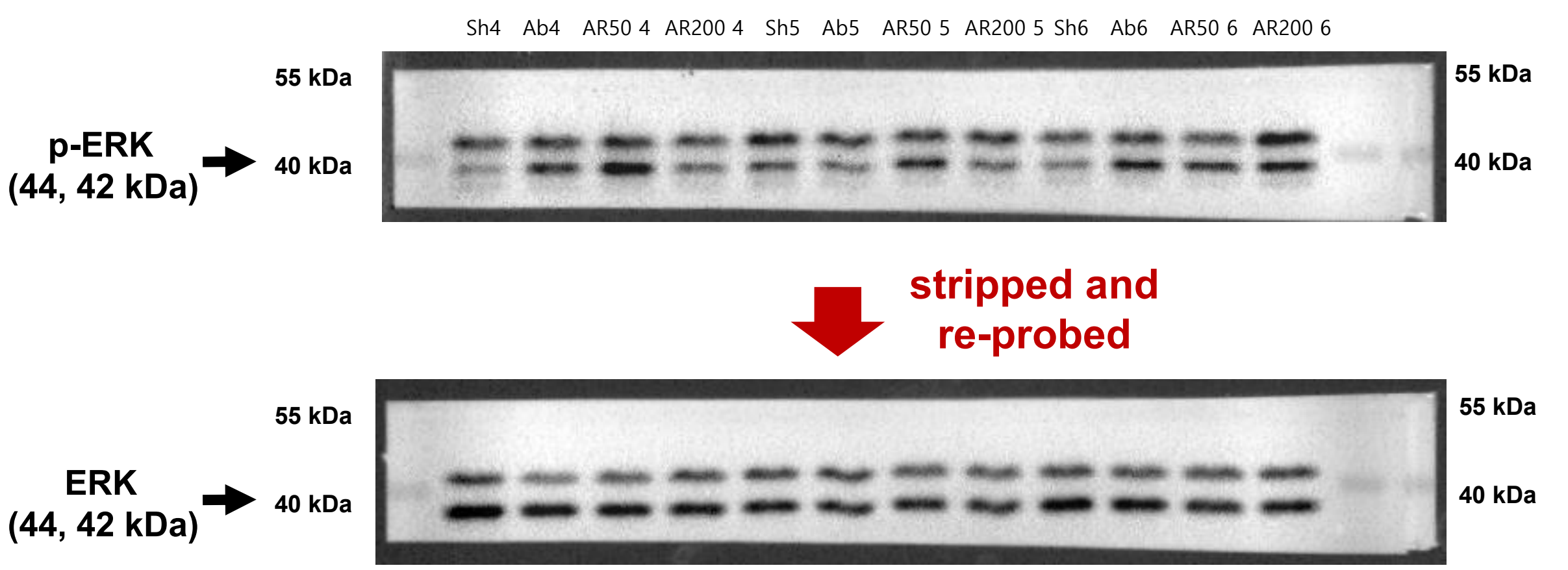

Blot images in Fig. 6

Code in blot images	
Sh (n=6)	Sham
Ab (n=6)	AβO
AR50 (n=6)	AβO+AR 50 mg/kg
AR200 (n=6)	AβO+AR 200 mg/kg

p-PI3K/PI3K
(no. 1~6 in each group)

highlight the protein bands (used in this manuscript with molecular weight details)

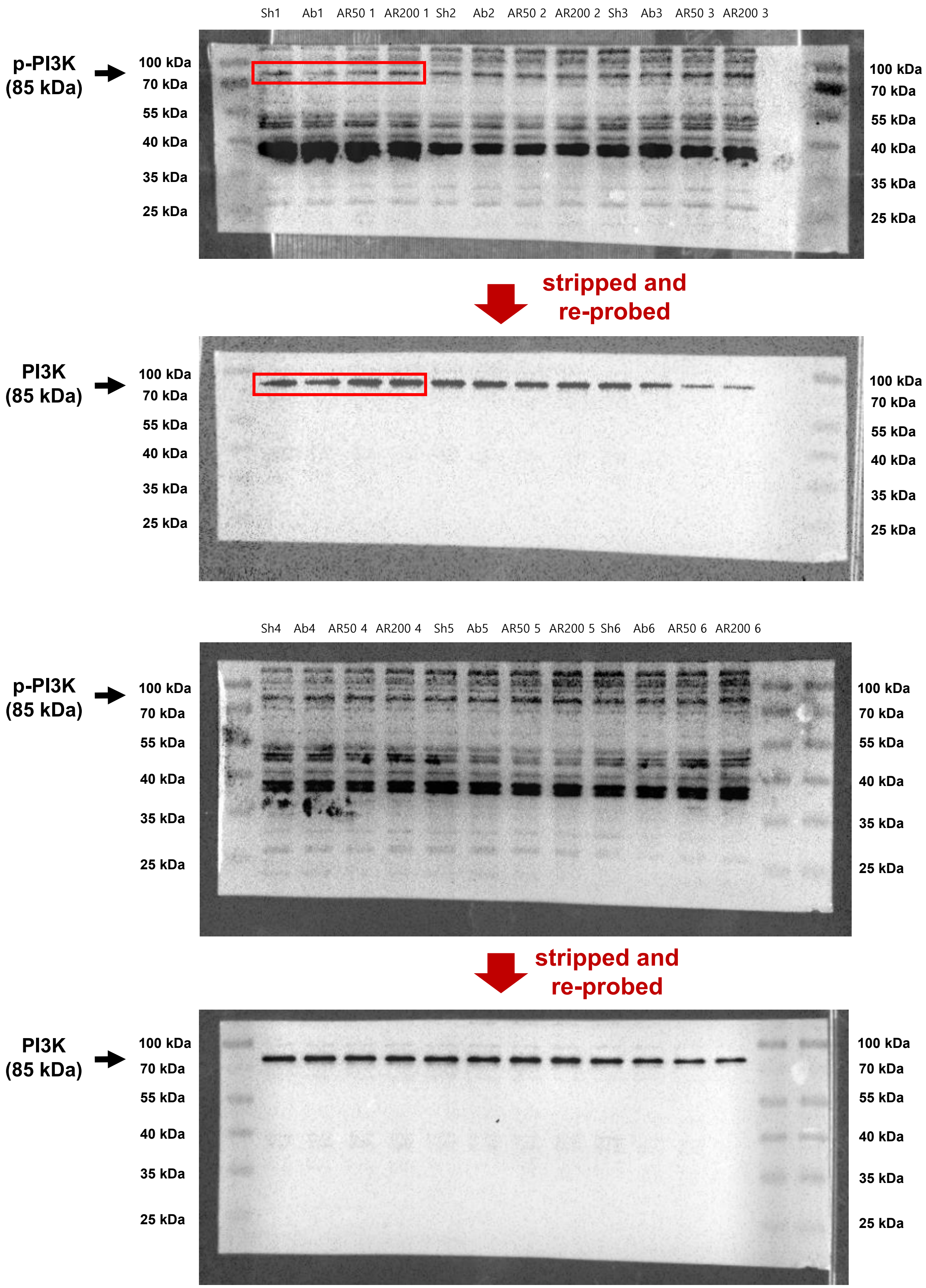

Blot images in Fig. 6

Code in blot images	
Sh (n=6)	Sham
Ab (n=6)	AβO
AR50 (n=6)	AβO+AR 50 mg/kg
AR200 (n=6)	AβO+AR 200 mg/kg

p-Akt/Akt
(no. 1~3 in each group)

highlight the protein bands (used in this manuscript with molecular weight details)

Sh1 Ab1 AR50 1 AR200 1 Sh2 Ab2 AR50 2 AR200 2 Sh3 Ab3 AR50 3 AR200 3

p-Akt (60 kDa) →

70 kDa
55 kDa
40 kDa
35 kDa
25 kDa
15 kDa

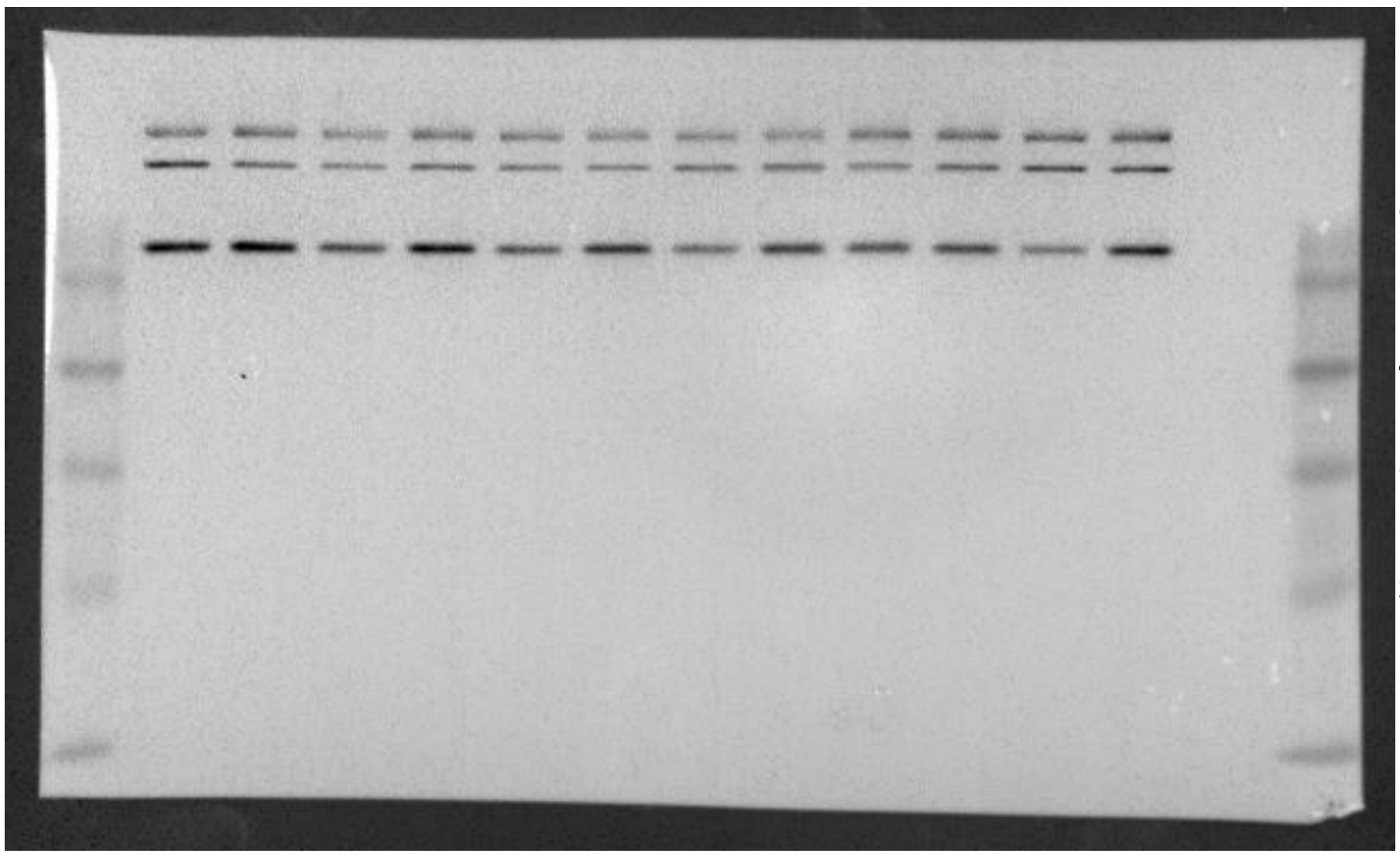

70 kDa
55 kDa
40 kDa
35 kDa
25 kDa
15 kDa

stripped and re-probed

Akt (60 kDa) →

70 kDa
55 kDa
40 kDa
35 kDa
25 kDa
15 kDa

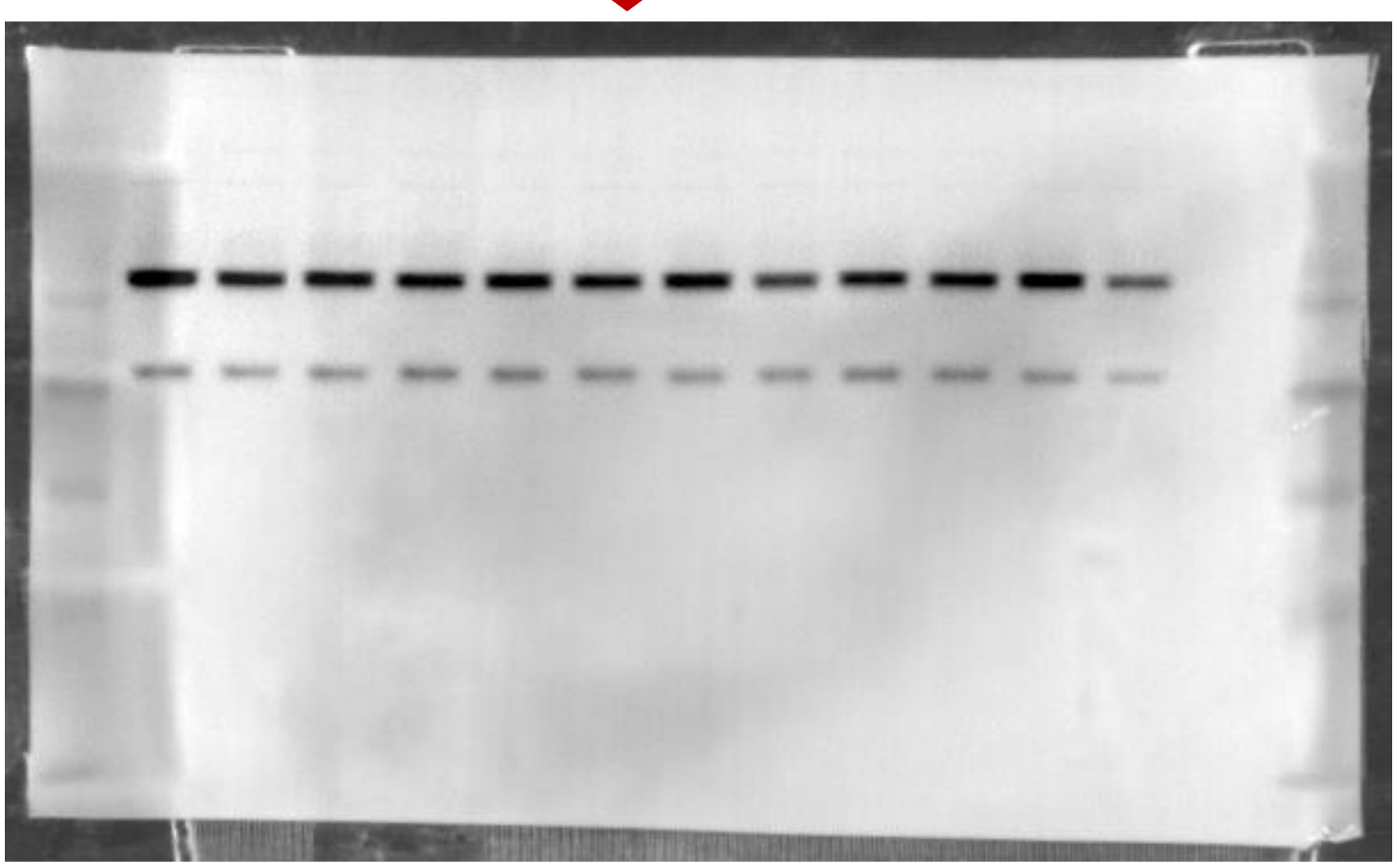

70 kDa
55 kDa
40 kDa
35 kDa
25 kDa
15 kDa

Blot images in Fig. 6

Code in blot images	
Sh (n=6)	Sham
Ab (n=6)	AβO
AR50 (n=6)	AβO+AR 50 mg/kg
AR200 (n=6)	AβO+AR 200 mg/kg

p-Akt/Akt
(no. 4~6 in each group)

highlight the protein bands (used in this manuscript with molecular weight details)

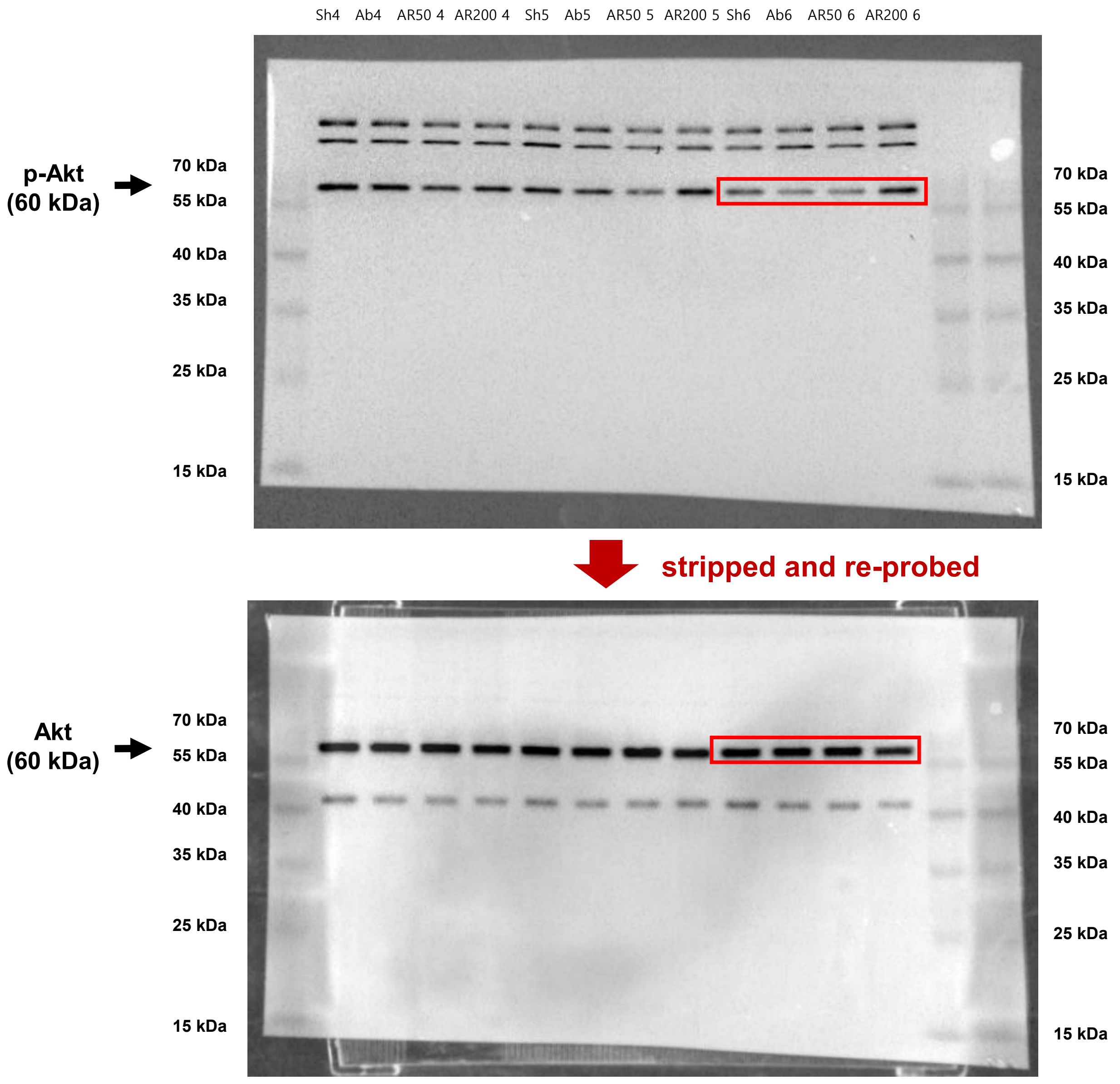

Blot images in Fig. 6

Code in blot images	
Sh (n=6)	Sham
Ab (n=6)	AβO
AR50 (n=6)	AβO+AR 50 mg/kg
AR200 (n=6)	AβO+AR 200 mg/kg

p-GSK-3β (ser9)/GSK-3β (no. 1~3 in each group)

highlight the protein bands (used in this manuscript with molecular weight details)

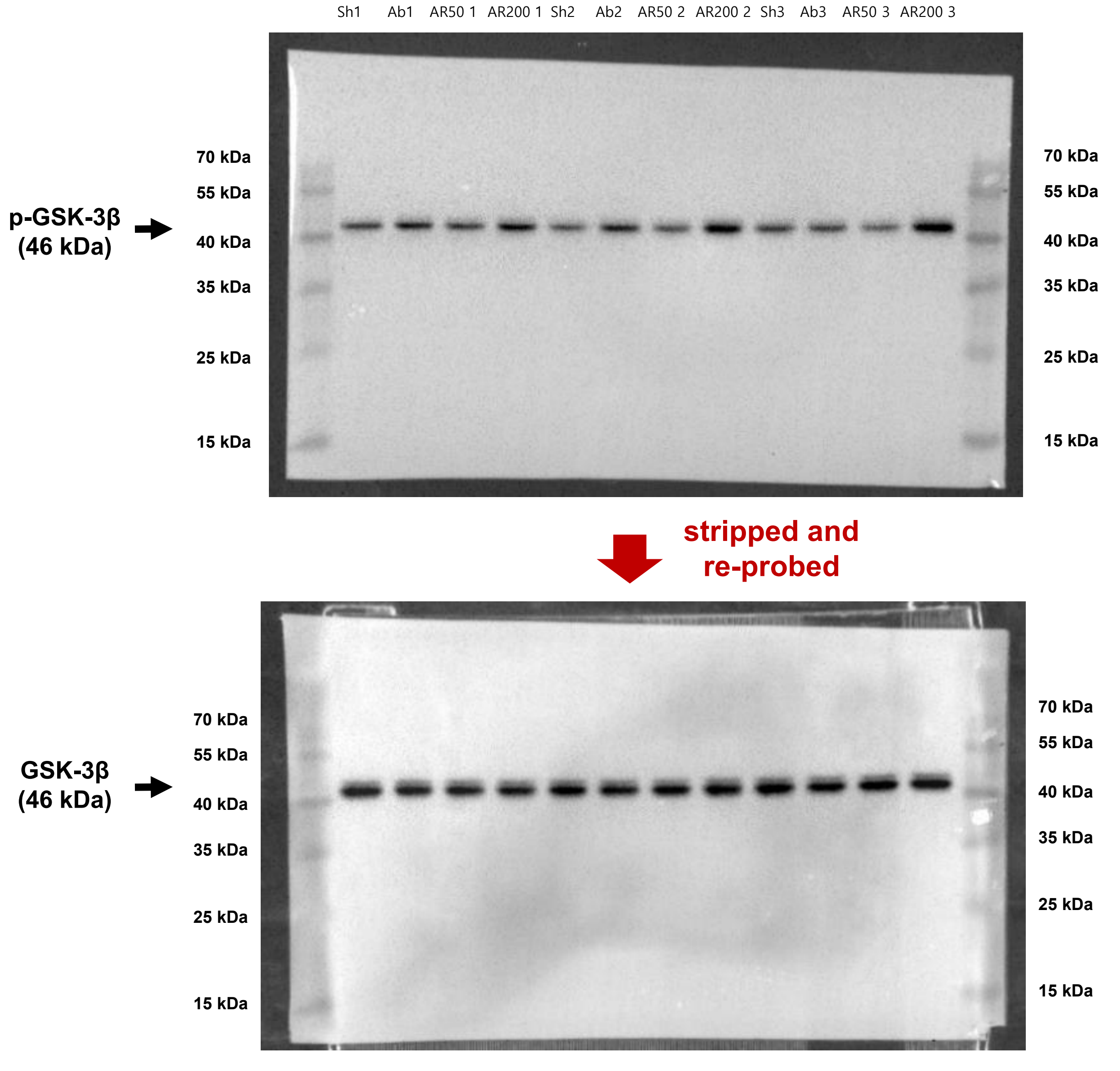

Blot images in Fig. 6

Code in blot images	
Sh (n=6)	Sham
Ab (n=6)	AβO
AR50 (n=6)	AβO+AR 50 mg/kg
AR200 (n=6)	AβO+AR 200 mg/kg

p-GSK-3β (ser9)/GSK-3β (no. 4~6 in each group)

highlight the protein bands (used in this manuscript with molecular weight details)

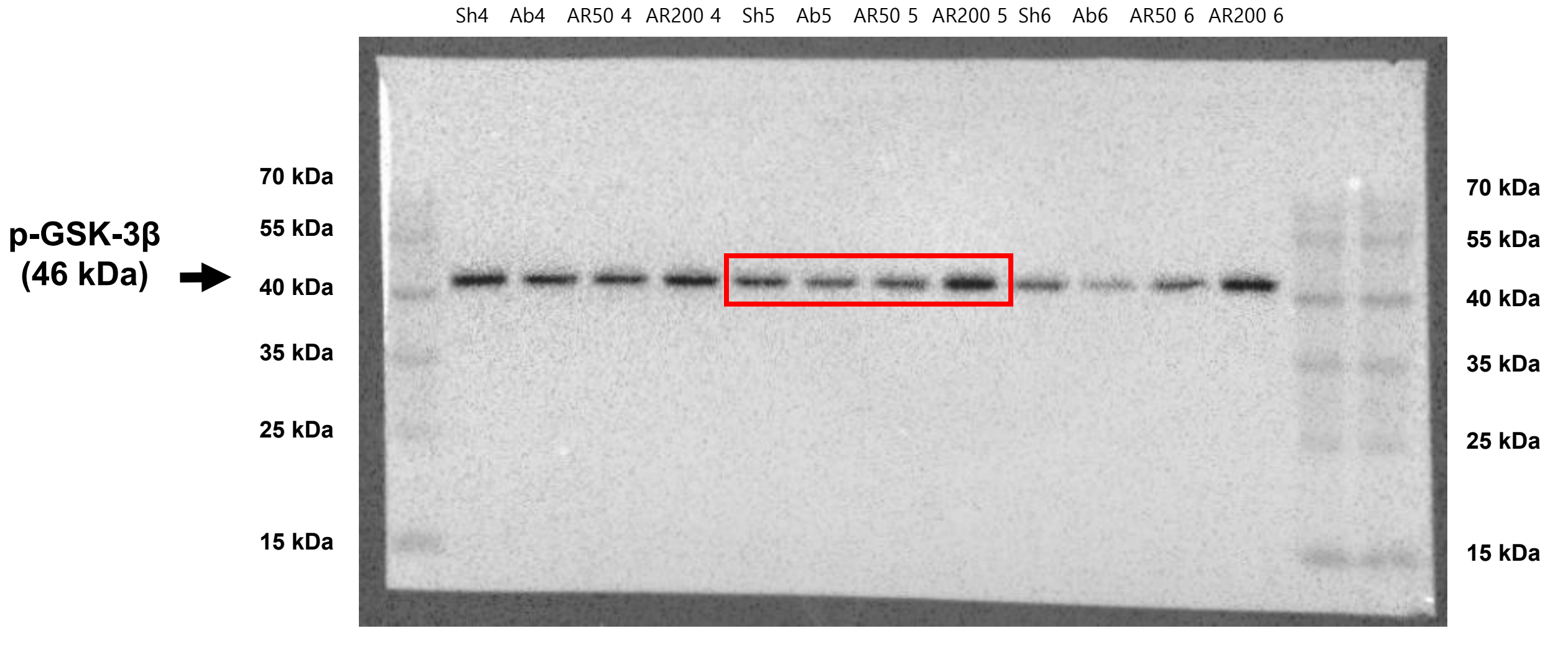

stripped and re-probed

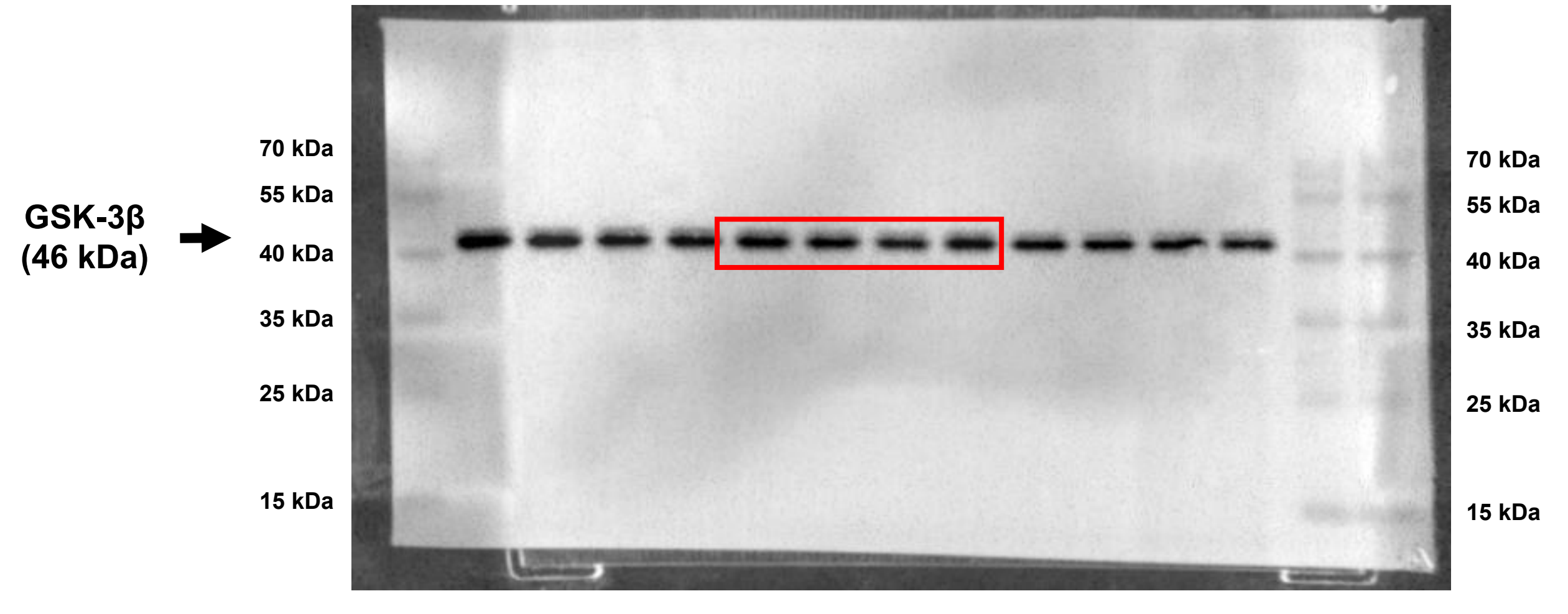

Blot images in Fig. 6

Code in blot images	
Sh (n=6)	Sham
Ab (n=6)	AβO
AR50 (n=6)	AβO+AR 50 mg/kg
AR200 (n=6)	AβO+AR 200 mg/kg

Cleaved caspase 3, Bax (no. 1~3 in each group)

highlight the protein bands (used in this manuscript with molecular weight details)

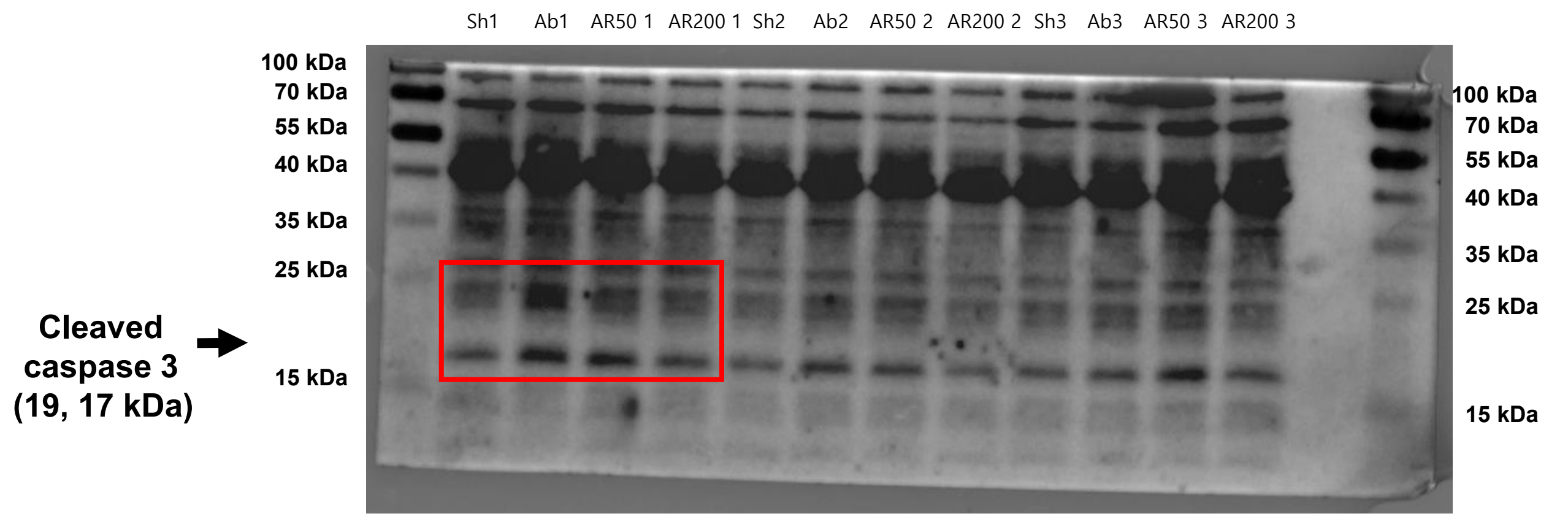

The membrane was subsequently cut and separately probed for Bax and β-actin.

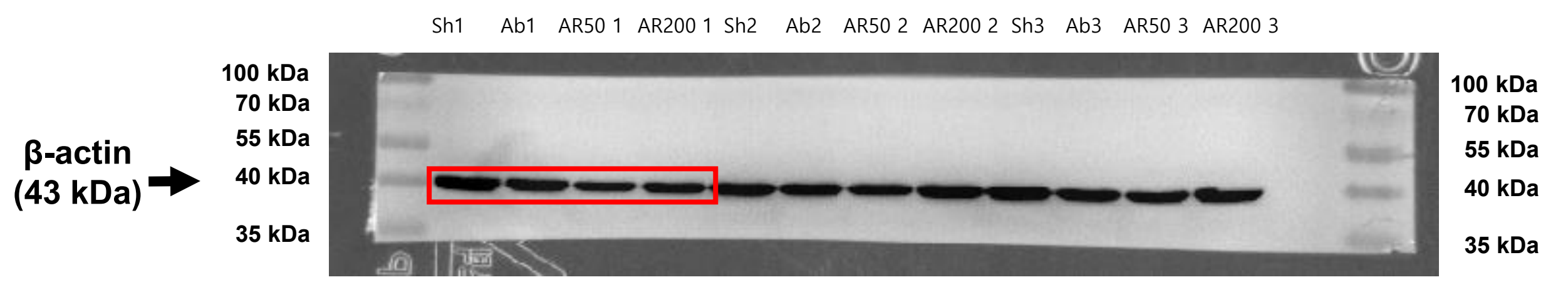

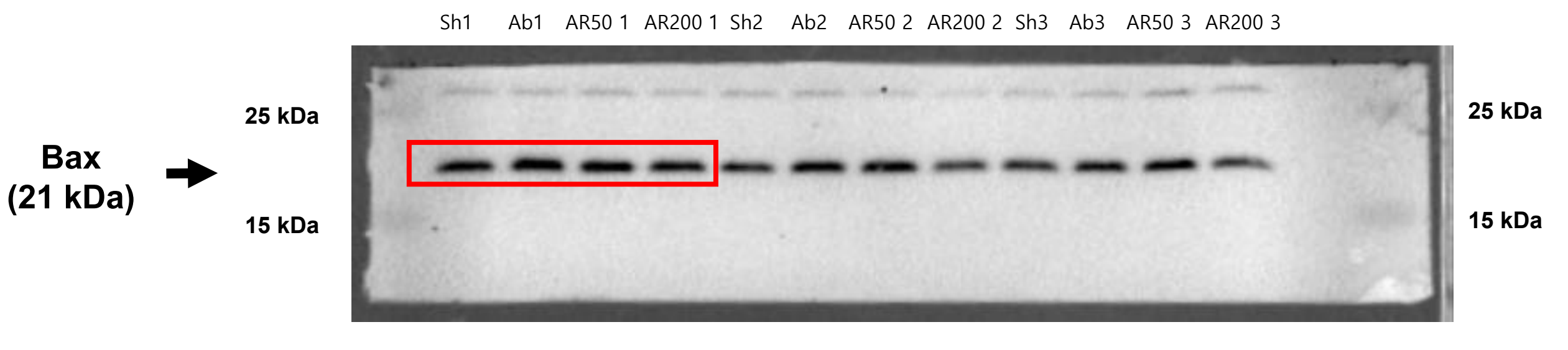

Blot images in Fig. 6

Code in blot images	
Sh (n=6)	Sham
Ab (n=6)	AβO
AR50 (n=6)	AβO+AR 50 mg/kg
AR200 (n=6)	AβO+AR 200 mg/kg

Cleaved caspase 3, Bax (no. 4~6 in each group)

highlight the protein bands (used in this manuscript with molecular weight details)

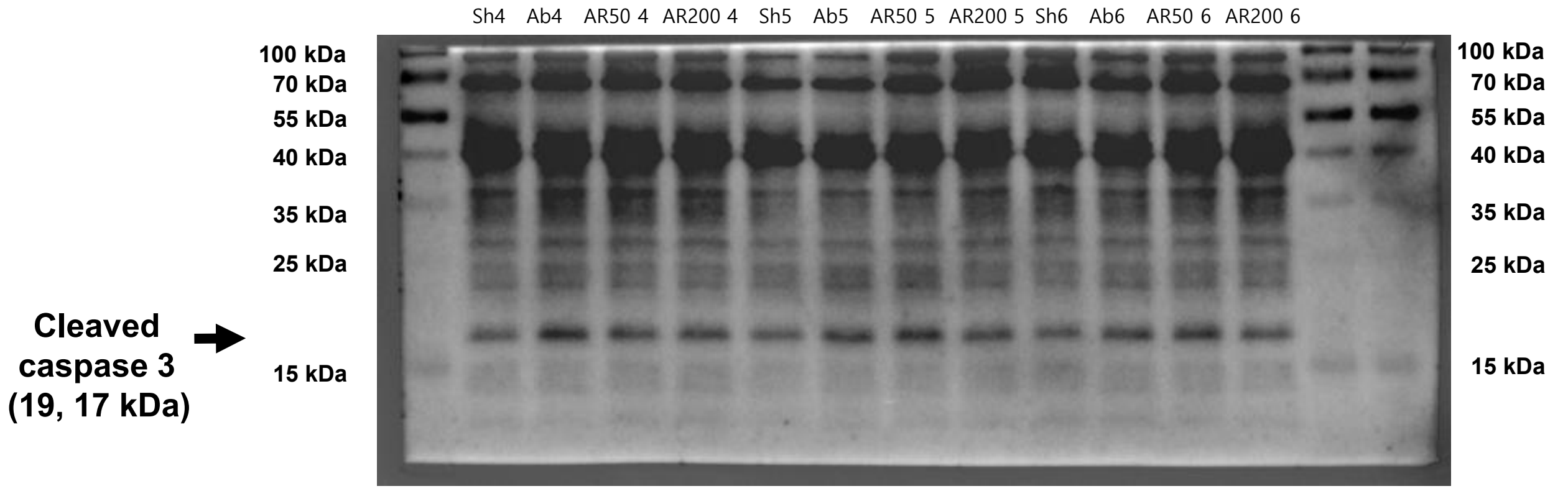

The membrane was subsequently cut and separately probed for Bax and β-actin.

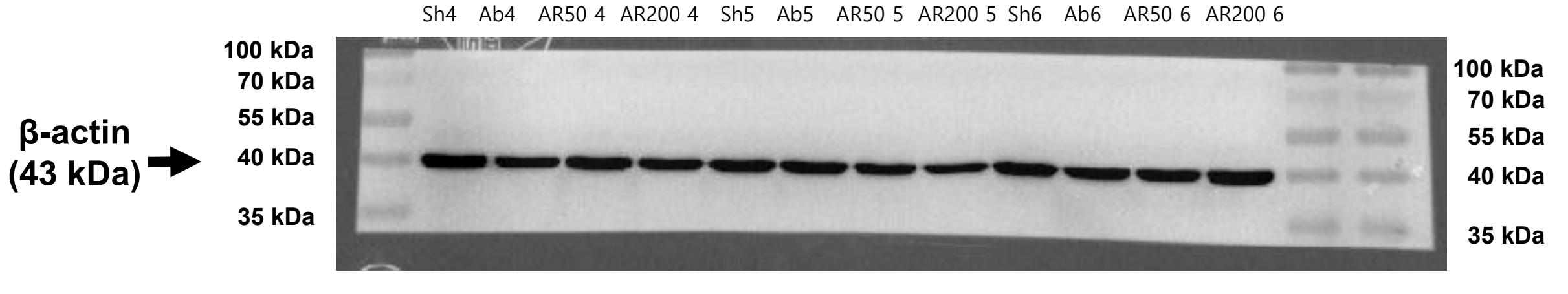

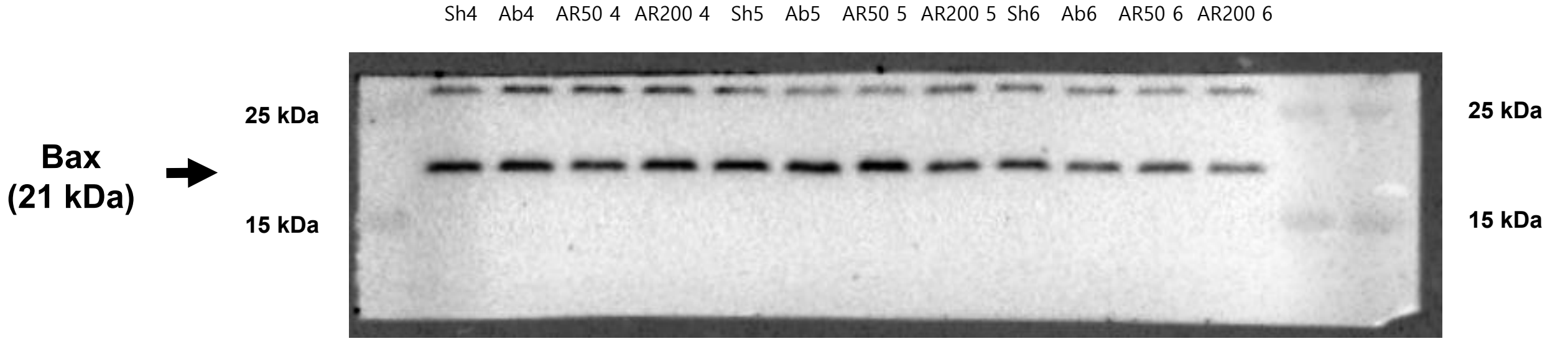

Blot images in Fig. 7

Cleaved caspase 3
(no. 1~6 in each group)

highlight the protein bands (used in this manuscript with molecular weight details)

Code in blot images	
C (n=4)	Control
Ab (n=4)	AβO
AbAL (n=4)	AβO + AR 30 μg/mL
AbAH (n=4)	AβO + AR 300 μg/mL

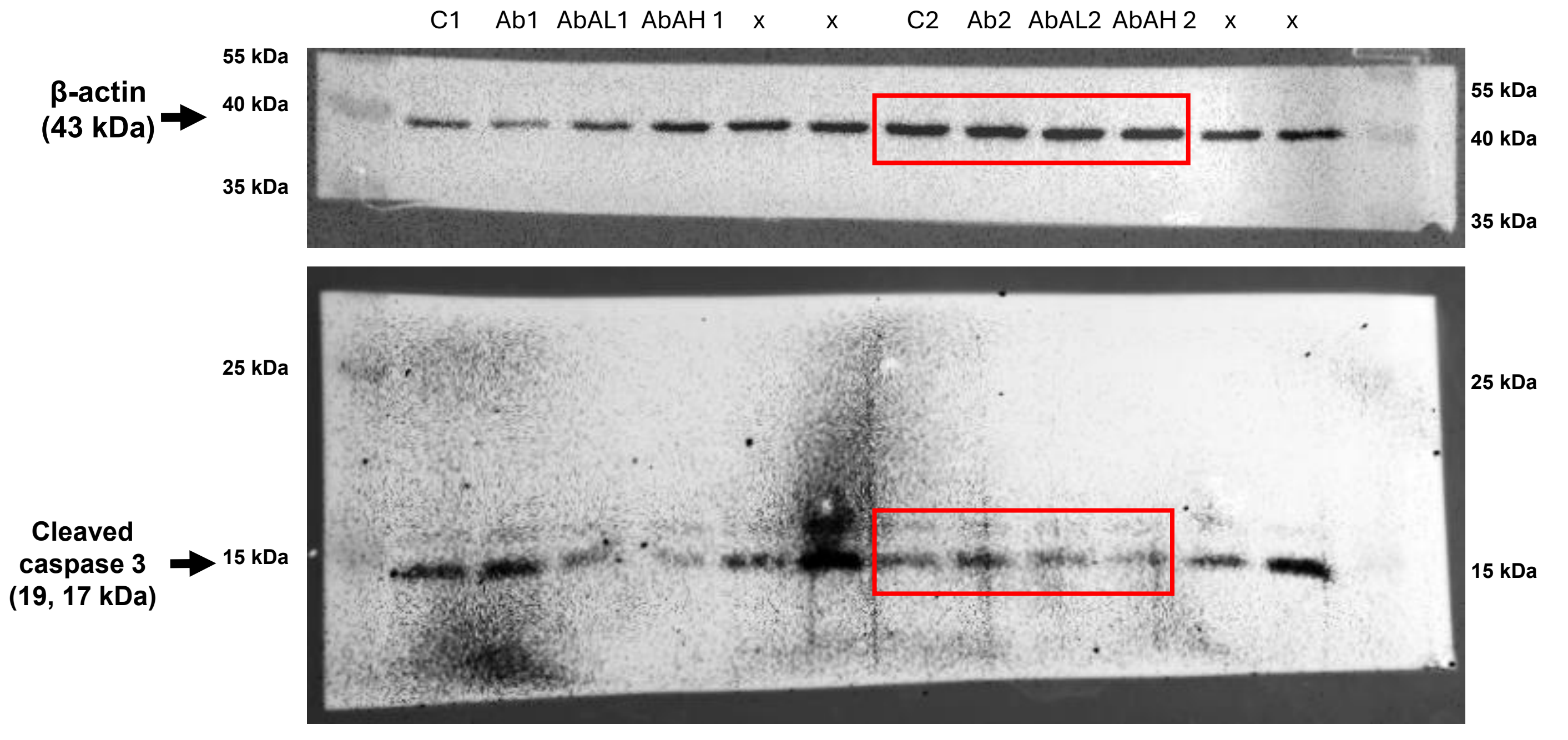

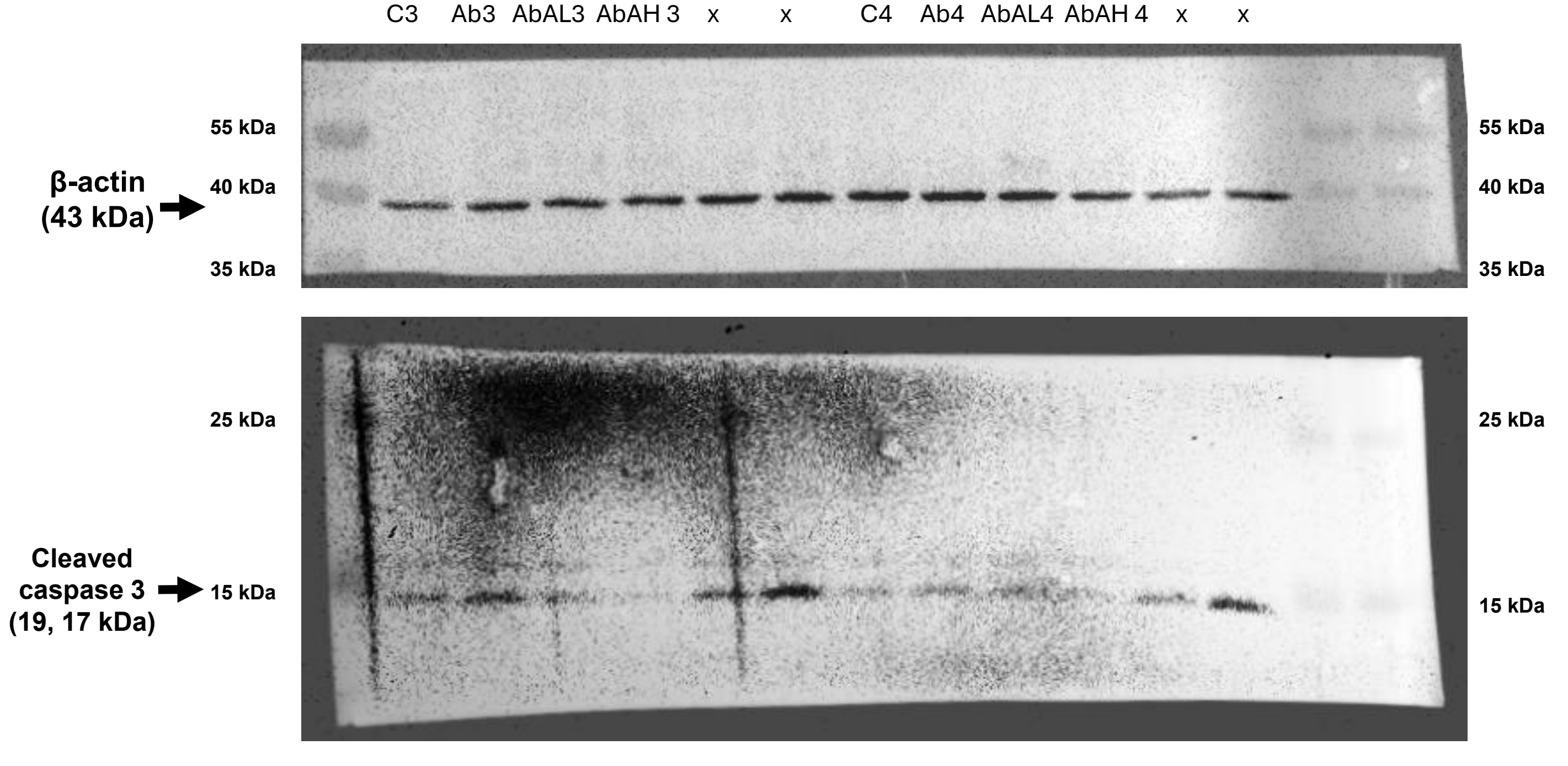

Blot images in Fig. 7

Code in blot images	
C (n=4)	Control
Ab (n=4)	AβO
AbAL (n=4)	AβO + AR 30 μg/mL
AbAH (n=4)	AβO + AR 300 μg/mL

p-GSK-3β (ser9)/GSK-3β (no. 1~6 in each group)

highlight the protein bands (used in this manuscript with molecular weight details)

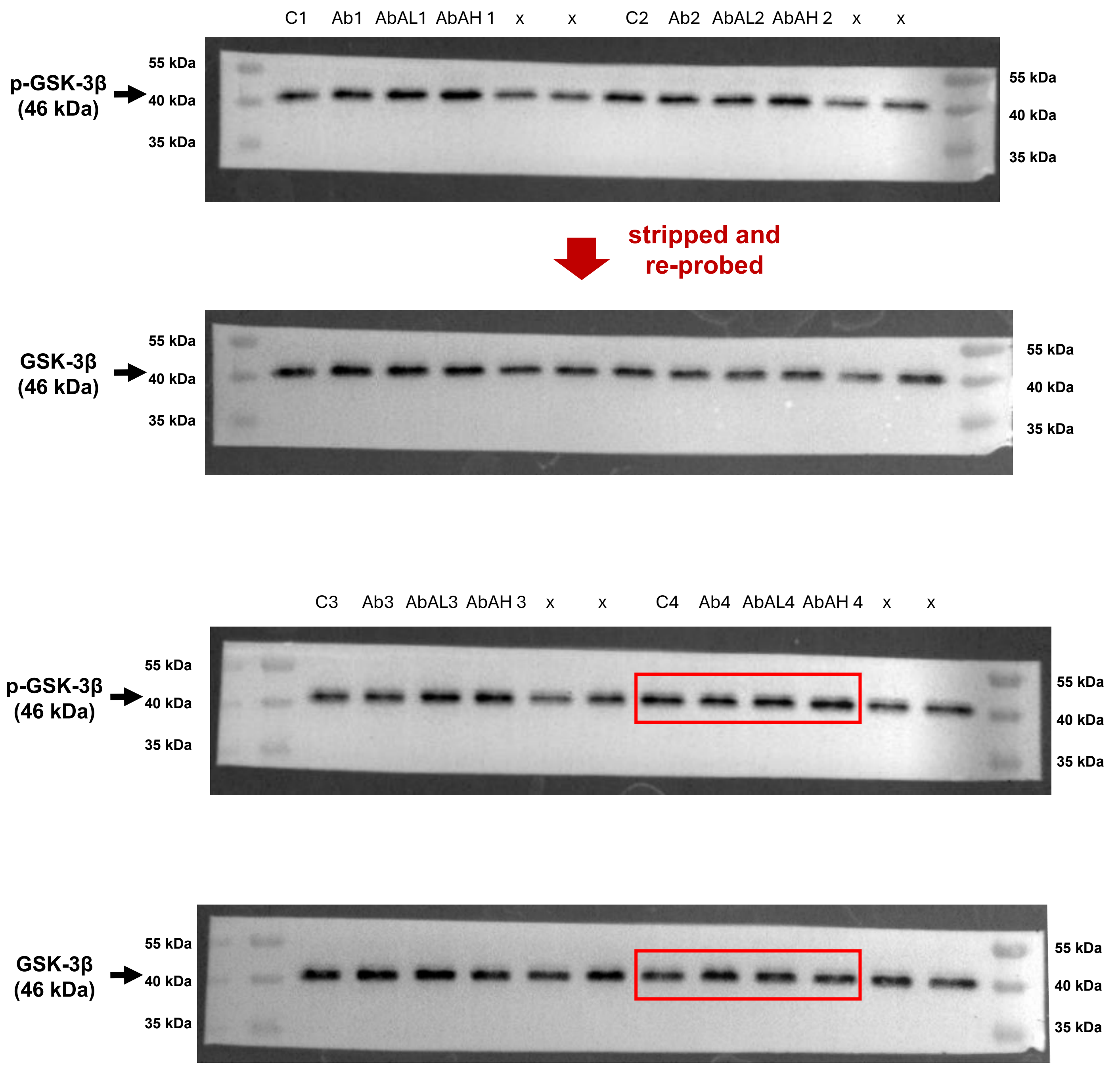

Supplement: Supplementary file 1 [file DataSheet1.pdf]
