# Supplementary material for: Alismatis Rhizoma prevents AβO-induced neuronal cell death and synaptic loss in Alzheimer’s disease models
Source: Front Pharmacol. 2026 Sep 4;17:1898356. doi: 10.3389/fphar.2026.1898356 (PMC13586005; doi:10.3389/fphar.2026.1898356)
Supplement: Supplementary file 2 [file Supplementaryfile1.docx]

Supplementary Material

**Supplementary Table S1. Calibration curves and quantitative results**

| **Analyte** | **Retention time**  **(min)** | **Calibration range**  **(μg/mL)** | **Regression equation** | **R²** | **Content (mg/g)** |
| --- | --- | --- | --- | --- | --- |
| Alisol A | 8.377 | 3.91–250 | Y = 9.3027X − 22.056 | 0.9995 | 0.235 ± 0.010 |
| Alisol B 23-acetate | 17.046 | 3.91–250 | Y = 9.6141X + 13.861 | 0.9993 | 0.504 ± 0.031 |

Data for compound contents are presented as the mean ± SD of four independent determinations. X represents the concentration of the standard compound (μg/mL), and Y represents the corresponding HPLC peak area.

**Supplementary Figure S1. Standard curves of Alisol A and Alisol B 23-acetate**

Supplementary Figure S1. Calibration curves of alisol A and alisol B 23-acetate. Calibration curves for alisol A (A) and alisol B 23-acetate (B) were constructed over the concentration range of 3.91–250 μg/mL. X represents the concentration of each reference standard, and Y represents the corresponding HPLC peak area.
